# Comparison of the efficacy and safety of Landiolol and Esmolol in critically ill patients: a propensity score-matched study

**DOI:** 10.1186/s13613-024-01418-8

**Published:** 2025-01-12

**Authors:** Xiang Si, Hao Yuan, Rui Shi, Wenliang Song, Jiayan Guo, Jinlong Jiang, Tao Yang, Xiaoxun Ma, Huiming Wang, Minying Chen, Jianfeng Wu, Xiangdong Guan, Xavier Monnet

**Affiliations:** 1 Critical Care Medicine, The First Affiliated Hospital of Sun Yat-Sen University, Guangzhou, China; 2 Service de Médecine Intensive-Réanimation, Hôpital de Bicêtre, DMU CORREVE, Inserm UMR S_999, FHU SEPSIS, Groupe de Recherche Clinique CARMAS, Université Paris-Saclay, AP-HP, Le Kremlin-Bicêtre, France; 3 Guangdong Clinical Research Centre for Critical Care Medicine, Guangzhou, 510080 China

**Keywords:** Beta-blocker, Heart rate, Hemodynamics, Mortality, Cardiac output, Septic shock

## Abstract

**Background:**

Excessive tachycardia is associated with impaired hemodynamics and worse outcome in critically ill patients. Previous studies suggested beneficial effect of β-blockers administration in ICU patients, including those with septic shock. However, comparisons in ICU settings are lacking. Our study aims to compare Landiolol and Esmolol regarding heart rate control and hemodynamic variables in general ICU patients.

**Methods:**

This retrospective, observational study was conducted in a 56-bed ICU at a university hospital. A propensity score matching (PSM) was employed to balance baseline differences. Generalized estimating equations (GEE) were used to compare heart rate between two drugs. The primary outcome was heart rate control, while secondary outcomes included hemodynamic response, hospital length of stay (HLOS) and ICU length of stay (ICULOS).

**Results:**

From June 2016 to December 2022, 438 patients were included after PSM, (292 in the Esmolol group and 146 the in Landiolol group). Baseline heart rate was similar between groups (Landiolol:120.0 [110.2, 131.0] bpm vs. Esmolol:120.0 [111.0, 129.0] bpm, *p* = 0.925). During 72 h. of β-blocker infusion, Landiolol reduced heart rate by 4.7 (1.3, 8.1) bpm, more than Esmolol (*p* = 0.007), while preserving a comparable proportion of patients able to stabilize vasopressor doses within the first 24 h. (82.9 vs. 80.8%, respectively, *p* = 0.596). Norepinephrine doses and lactate levels were similar between groups over 72 h., while the Landiolol group exhibited notably higher minimal ScvO_2_ levels (72% [63%, 78%] vs 68% [55%, 73%], respectively, *p* = 0.006) and a lower maximal PCO2 gap compared to the Esmolol group (7.0 [6.0, 9.0] vs. 8.0 [6.0, 10.0] mmHg, respectively, *p* = 0.040). Patients in the Landiolol group were observed to experience shorter HLOS than patients in the Esmolol group (26.5 [13.0, 42.0] *vs* 30.0 [17.0, 47.2] days, respectively, *p* = 0.044) and ICULOS (4.9 [2.8, 10.0] *vs.*6.7 [3.4, 13.1] days, respectively, *p* = 0.011).

**Conclusion:**

Landiolol provides superior heart rate control in critically ill patients with tachycardia compared to Esmolol, without increasing vasopressor requirements during the first 24 h. Findings from ScvO_2_ levels and PCO_2_ gap suggest that Landiolol may exert less impact on cardiac output than Esmolol. Further studies, incorporating comprehensive hemodynamic monitoring, are warranted to clarify the clinical implications of heart rate control with β-blockers in ICU patients with tachycardia.

**Supplementary Information:**

The online version contains supplementary material available at 10.1186/s13613-024-01418-8.

## Background

Critically ill patients often experience excessive sympathetic stimulation due to high levels of circulating catecholamines, leading to elevated blood pressure, hypermetabolic state, and increased heart rate. Tachycardia, occurring primarily or secondarily, is commonly encountered in patients admitted to intensive care unit (ICU) [1] and has been identified as an independent risk factor for mortality when heart rates exceed 100 beats per minute (bpm) [2, 3]. Beta-adrenergic blockers (β-blockers), traditionally used in the cardiovascular therapeutic armamentarium, have garnered attention for their potential benefits in modulating pathophysiological responses in critically ill patients, such as weakening of inflammatory cytokines, adjustment of metabolic dysregulation, and regulation of the immune system, etc. [4–6].

Generally, β-blockers exert their effects by preventing and/or modulating the β-adrenergic responses through several subtypes of G-protein-coupled β-adrenergic receptors expressed on cell membranes [7]. This could dampen the effects of catecholamines such as epinephrine and norepinephrine (NE) [7]. Some studies suggested that intravenous selective short-acting β-blocker agents, for example, Esmolol, may offer potential clinical benefits in critically ill patients, including those with septic shock [8–10]. Landiolol, a selective ultra-short acting agent compared to Esmolol, was reported to have higher cardioselectivity [11], less potent negative inotropy [12], and a smaller blood pressure-lowering effect [13] in animal models and healthy volunteers. However, the two most recent randomized controlled trials, the STRESS-L study [14] and the Landi-SEP study [15], reported that in patients with septic shock, while Landiolol effectively reduced heart rate, it did not reduce organ failure nor improved outcomes compared with standard care. Furthermore, evidence comparing Landiolol and Esmolol is lacking in real-world ICU settings.

Therefore, the objective of this study was to compare the efficacy and safety of Landiolol and Esmolol in treating tachycardia and their potential impact on the hemodynamics of ICU patients.

## Methods

This is a single-center, retrospective, observational study conducted in a general ICU with 56 beds in a tertiary hospital (First Affiliated Hospital, Sun Yat-sen University, Guangzhou, China). Ethics approval was obtained from the Independent Ethics Committee for Clinical Research and Animal Trials of Hospital (approval No. [2020] 266-1). A waiver of informed consent was granted for collection of existing data. The study has been registered in the China Clinical Trial Registry (ChiCTR2300077270) and was performed following the Strengthening the Reporting of Observational Studies in Epidemiology (STROBE) statement [16].

### Patients

We included patients hospitalized in our ICU, consisting of approximately 80% surgical and 20% medical patients. The decision to prescribe β-blockers was made by attending physicians based on drug pharmacodynamics and the Chinese Guidelines for Sepsis/Septic Shock Management (2014) to control persistent tachycardia [17]. Additionally, patients who underwent surgery for aortic dissection or aneurysm, requiring strict heart rate control, were also managed with β-blockers. The selection of Landiolol or Esmolol was primarily based on physician preference, given the absence of specific guidelines recommending one agent over the other. Patients satisfying all the following criteria from June 2016 to December 2022 were included in the study: i) tachycardia with a heart rate ≥100 bpm before administration of Esmolol or Landiolol; ii) infusion of Esmolol or Landiolol within 72 h. of ICU admission; and iii) administration of Esmolol or Landiolol without any other anti-arrhythmic drugs. Only the first hospitalization was included among patients with multiple ICU admissions meeting the aforementioned criteria. Exclusion criteria were: i) age <18 y.o., ii) pregnancy, iii) absence of data on heart rate and the β-blockers injection time, and iv) history of longer-acting, nonspecific β-blockers prior to ICU admission.

### Measurements

Data extracted included heart rate, systolic (SAP), diastolic (DAP) and mean (MAP) arterial pressure, vasopressor dosage, β-blockers infusion time and dosage, lactate, central venous oxygen saturation (ScvO_2_) and the veno-arterial difference in carbon dioxide partial pressure (PCO_2_ gap) at 3rd, 6th, 8th, 12th, 24th, 48th and 72nd hrs. after initiating β-blockers. Sepsis was defined as a documented infection accompanied by an acute increase in the Sequential Organ Failure Assessment (SOFA) score of 2 points or more. Septic shock was defined according to current recommendations of Sepsis-3 [18]. Vasopressor stability was defined as maintaining or de-escalating vasopressor doses to achieve a MAP ≥65 mmHg at 24 h. after initiating β-blocker infusion [19]. The primary outcome was the reduction in heart rate within the first 72 h. of drug infusion. Secondary outcomes included: i) hemodynamic stability during the infusion period; ii) ICU and 60-day mortality; iii) hospital length of stay (HLOS) and ICU length of stay (ICULOS).

### Statistical analysis

Quantitative variables were expressed as medians with interquartile ranges, while categorical variables were presented as frequencies and percentages. The Wilcoxon rank sum test was used for continuous variables, and the chi-square or Fisher’s exact test for categorical variables. Propensity score matching (PSM) was applied to balance baseline characteristics between the Landiolol and Esmolol groups [20]. Nearest neighbor matching was performed at a 1:2 ratio without replacement, with a caliper width of 0.02. Baseline heart rate, MAP, NE dose, sex, age, mechanical ventilation, ratio of arterial partial pressure of oxygen to fraction of inspired oxygen (PaO_2_/FiO_2_), arterial lactate, sepsis, diabetes, hypertension, tumor history, surgery, acute kidney injury (AKI) stage according to Kidney Disease: Improving Global Outcomes classification [21], and SOFA score prior to β-blockers initiation were included in the logistic model to calculate propensity scores. The standardized mean difference (SMD < 0.1) was calculated for these variables, and missing values were imputed using the random forest method.

Mean heart rate during the 72-h. period was calculated using Matthews’ method [22]. Heart rate, blood pressure and NE dosage as dependent variables were analyzed by generalized estimating equations (GEE) model [23], which accounted for the correlation between repeated measurements. The interaction effect was assessed by incorporating an interaction term between the ‘group’ and ‘time’ variables into the regression model. The effects of Landiolol and Esmolol on HLOS and ICULOS were also evaluated.

Prespecified subgroup analysis included septic shock patients, postoperative patients, elderly patients, and those with a baseline heart rate ≥120 bpm. Heart rate reduction over 72 h. was assessed using GEE models across all subgroups. ICU mortality, 60-day mortality, ICULOS and HLOS were analyzed, with results displayed in the forest plot.

Two sensitivity analysis were conducted: i). patients who remained in the ICU for more than 24 h. after the initiation of β-blockers infusion were selected to estimate heart rate difference between the groups; ii). different seeds were applied for imputing missing values to verify the robustness of the primary results.

All statistical analysis were performed with R 4.2.2 (R Foundation for Statistical Computing, Vienna, Austria), with a two-sided *p* < 0.05 considered statistically significant.

## Results

### Patient characteristics

**Fig. 1:**
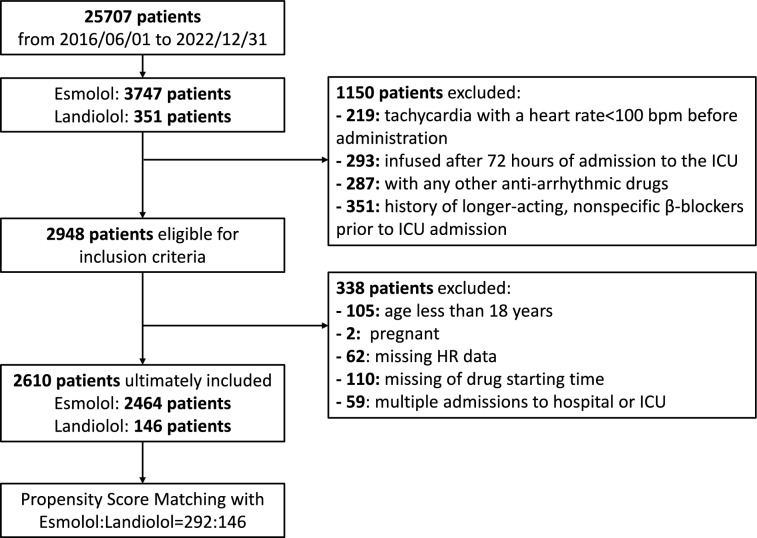
Flowchart inclusion procedure

The detailed flow chart is illustrated in Fig. 1. Among the 2,610 patients meeting the inclusion criteria, 2,464 received Esmolol, while 146 were administered Landiolol. After PSM, 438 patients were included in the study, comprising 292 in the Esmolol group and 146 in the Landiolol group. Notably, 40% of these patients were admitted for sepsis or septic shock, and atrial fibrillation was observed in 10% of the cohort. The baseline characteristics before and after PSM are summarized in Table 1 and Table S1. The primary indication for β-blocker use was the reduction of persisting tachycardia in all kinds of patients. This included a strict control of heart rate in patients who underwent surgery for aortic dissection or aneurism.

**Table 1:** Baseline characteristics after the propensity score matching

Characteristic	Overall, N = 438	Esmolol, N = 292	Landiolol, N = 146	SMD^1^	*p*-value^2^
Age, median (IQR)	62 (50.0, 71)	62 (50.8, 71)	61 (50.0, 71)	−0.04	0.747
Female, n (%)	150 (34.2%)	101 (34.6%)	49 (33.6%)	0.02	0.831
Primary diagnosis, n (%)					
Sepsis/Septic shock	175 (40.0%)	118 (40.4%)	57 (39.0%)	0.03	0.783
Sepsis	53 (12.1%)	38 (13.0%)	15 (10.3%)		
Septic shock	122 (27.9%)	80 (27.4%)	42 (28.8%)		
Post-operative	177 (40.4%)	115 (39.4%)	62 (42.5%)	−0.06	0.535
Abdominal surgery	88 (20.1%)	58 (19.9%)	30 (20.5%)		
Trauma	44 (10.0%)	23 (7.9%)	21 (14.4%)		
Others	45 (10.3%)	34 (11.6%)	11 (7.5%)		
Medical	86 (19.6%)	59 (20.2%)	27 (18.5%)	0.04	0.671
Acute respiratory failure	28 (6.4%)	14 (4.8%)	14 (9.6%)		
Others	58 (13.2%)	45 (15.4%)	13 (8.9%)		
Atrial fibrillation, n (%)	43 (9.8%)	28 (9.6%)	15 (10.3%)	−0.02	0.820
Hemodynamic variables					
Heart rate, median (IQR)	120.0 (111.0, 129.7)	120.0 (111.0, 129.0)	120.0 (110.3, 131.0)	−0.02	0.925
MAP, median (IQR)	86.0 (77.2, 92.0)	87.0 (78.0, 93.0)	85.0 (77.0, 91.0)	0.08	0.134
Lactate, median (IQR)	1.7 (1.1, 2.8)	1.7 (1.0, 2.8)	1.8 (1.2, 2.6)	−0.10	0.330
Norepinephrine dose, median (IQR)	0.0 (0.0, 0.2)	0.0 (0.0, 0.2)	0.0 (0.0, 0.2)	0.08	0.245
β-blocker dose, median (IQR)	4.6 (2.0, 9.7)	5.5 (2.0, 12.8)	3.1 (2.2, 5.1)	0.64	<0.001
Time for initiating β-blocker infusion, median (IQR)	20.2 (10.7, 32.8)	20.0 (11.9, 32.4)	20.8 (9.2, 35.9)	−0.08	0.975
Subsequent oral β-blocker agent	173 (39.5%)	119 (40.8%)	54 (37.0%)	0.08	0.447
Organ function					
Baseline SOFA, median (IQR)	7.7 (6.0, 9.0)	7.7 (6.0, 9.0)	7.7 (6.0, 9.8)	−0.04	0.214
Mechanical ventilation, n (%)	291 (66.4%)	190 (65.1%)	101 (69.2%)	−0.09	0.391
PaO_2_/FiO_2_, median (IQR)	303.0 (210.0, 385.3)	305.0 (209.5, 388.1)	297.0 (211.3, 378.1)	0.05	0.666
AKI stage, n (%)				0.07	0.871
0	307 (70.1%)	202 (69.2%)	105 (71.9%)		
1	31 (7.1%)	20 (6.8%)	11 (7.5%)		
2	8 (1.8%)	6 (2.1%)	2 (1.4%)		
3	92 (21.0%)	64 (21.9%)	28 (19.2%)		
Diabetes, n (%)	85 (19.4%)	58 (19.9%)	27 (18.5%)	0.03	0.733
Hypertension, n (%)	145 (33.1%)	94 (32.2%)	51 (34.9%)	−0.06	0.566
Tumor, n (%)	219 (50.0%)	151 (51.7%)	68 (46.6%)	0.10	0.311

*AKI* acute kidney injury, *IQR* interquartile range, *MAP* mean artery pressure, *PaO*_*2*_*/FiO*_*2*_ ratio of arterial oxygen tension to fraction of inspired oxygen

^1^Standardized Mean Difference

^2^Wilcoxon rank sum test; Pearson’s Chi-squared test; Fisher’s exact test

### Heart rate

**Fig. 2:**
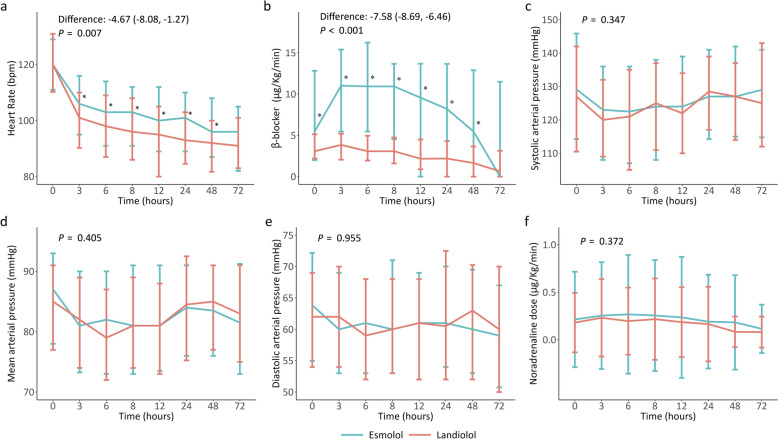
Trend of **a** Heart Rate, **b** β-blocker, **c** Systolic arterial pressure, **d** Mean arterial pressure, **e** Diastolic arterial pressure, **f** Norepinephrine doses during the first 72 h. of Esmolol and Landiolol in the whole population. * indicates a significant difference between Esmolol and Landiolol group at the specified time

The mean heart rate over the 72 h. was significantly lower in the Landiolol group, at 95.9 (87.8, 104.4) bpm, compared to 101.3 (93.8, 108.0) bpm in the Esmolol group (*p* < 0.001). According to the GEE model, Landiolol reduced heart rate by an additional 4.7 (1.3, 8.1) bpm (*p* = 0.007) compared to Esmolol during the 72-h. infusion (Fig. 2a). Furthermore, the proportion of patients with a heart rate <95 bpm throughout the infusion period was significantly larger in the Landiolol group compared to the Esmolol group (Table 2).

**Table 2:** Comparison of primary and secondary outcomes between Landiolol group and Esmolol group

Characteristic	Overall, N = 438	Esmolol, N = 292	Landiolol, N = 146	*p*-value^1^
Primary outcome				
Mean heart rate over 72 h (bpm), median (IQR)		101.3 (93.8, 108.0)	95.9 (87.8, 104.4)	<0.001
Proportion of patients with heart rate <95 bpm, n (%)				
3 h	117 (26.7%)	66 (22.6%)	51 (34.9%)	0.006
6 h	149 (34.0%)	88 (30.1%)	61 (41.8%)	0.015
8 h	157 (35.8%)	92 (31.5%)	65 (44.5%)	0.007
12 h	187 (42.7%)	112 (38.4%)	75 (51.4%)	0.009
24 h	220 (50.2%)	126 (43.2%)	94 (64.4%)	<0.001
48 h	277 (63.2%)	171 (58.6%)	106 (72.6%)	0.004
72 h	307 (70.1%)	195 (66.8%)	112 (76.7%)	0.032
Secondary outcome				
Vasopressor stability^2^, n (%)	360 (82.2%)	242 (82.9%)	118 (80.8%)	0.596
Hospital LOS, median (IQR)	29.0 (15.0, 44.8)	30.0 (17.0, 47.2)	26.5(13.0, 42.0)	0.044
ICULOS, median (IQR)	5.8 (3.0, 11.8)	6.7 (3.4, 13.1)	4.9 (2.8, 10.0)	0.011
Mechanical ventilation hours, median (IQR)	38.5 (2.5, 172.4)	36.0(1.2, 198.4)	39.6 (7.5, 123.1)	0.734

*ICU* intensive care unit, *IQR* interquartile range, *LOS* length of stay

^1^Pearson’s Chi-squared test; Wilcoxon rank sum test

^2^Vasopressor stability definition: maintaining or de-escalating vasopressor doses at 24 h. compared to the treatment start

### Other hemodynamic variables

The duration of β-blocker infusion over the 72 h. was comparable between the two groups, with Landiolol being infused for 33.0 (17.0, 68.8) hrs. vs. 33.0 (13.8, 62.0) hrs. for Esmolol (*p* = 0.134). The initial dosage of Landiolol was 3.1 (2.2, 5.1) μg/kg/min, compared to 5.5 (2.0, 12.8) μg/kg/min for Esmolol (*p* < 0.001). The subsequent doses at the 3rd, 6th, 8th, 12th, 24th, and 48th hrs. are shown in Fig. 2b.

The vasopressor stability at 24 h. after initiating either Esmolol or Landiolol infusion was similar between groups (82.9 vs. 80.8%, respectively, *p* = 0.596) (Table 2). During the 24-h. infusion period, MAP increased by 0.11 mmHg per hour in the Landiolol group (*p* = 0.087) and by 0.09 mmHg per hour in the Esmolol group (*p* = 0.082; *p* = 0.769 between groups), while NE doses decreased by 2.8 × 10⁻^3^ μg/kg/min (*p* = 0.121) and 3.5 × 10⁻^3^ μg/kg/min (*p* = 0.031; *p* = 0.785 between groups), respectively (Fig. 2d, f). No significant changes over time or differences between groups were observed in SAP, MAP or DAP, nor in NE dosages during the 72-h. period (Fig. 2c–f; Table S3).

**Fig. 3:**
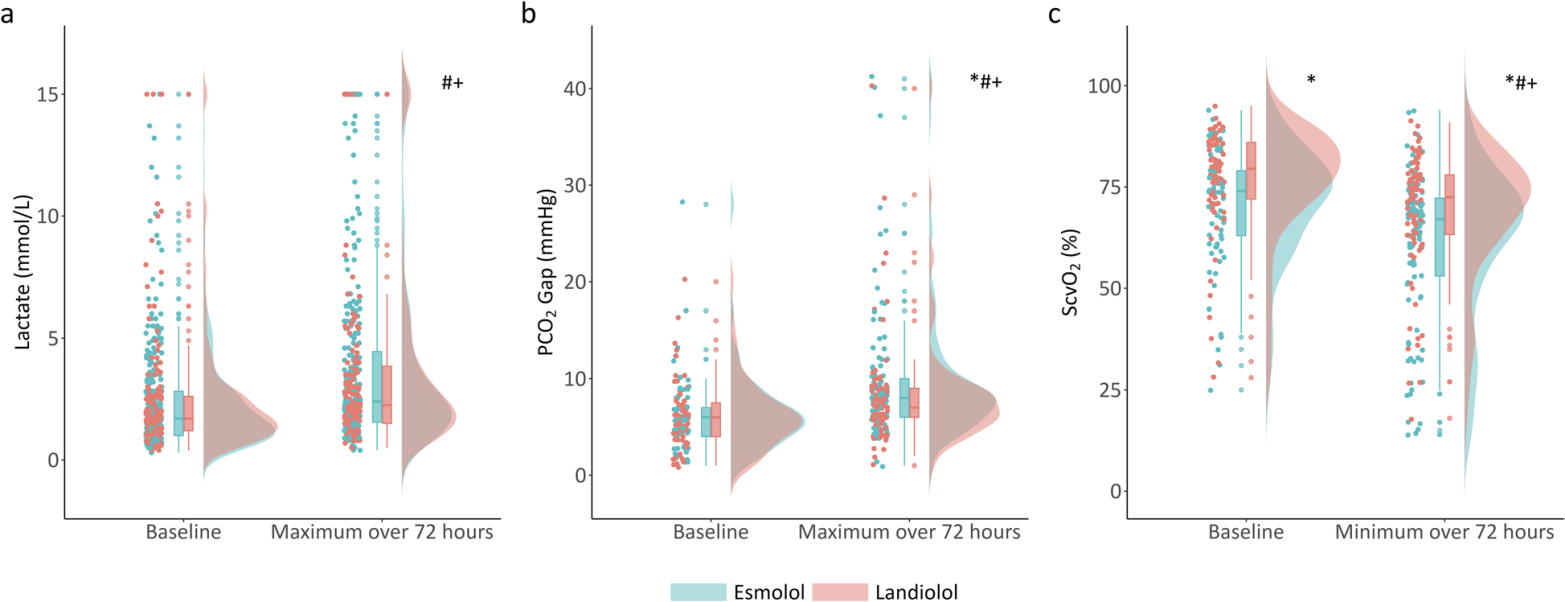
Comparison of lactate levels (**a**), PCO_2_ gap (**b**), and ScvO_2_ (**c**) at baseline and during β-blockers infusion among all the patients using raincloud plots. * indicates a significant difference between Esmolol and Landiolol group at baseline and over 72 h. # indicates a significant difference between baseline and over 72 h. in Esmolol group. + indicates a significant difference between baseline and over 72 h. in Landiolol group

**Fig. 4:**
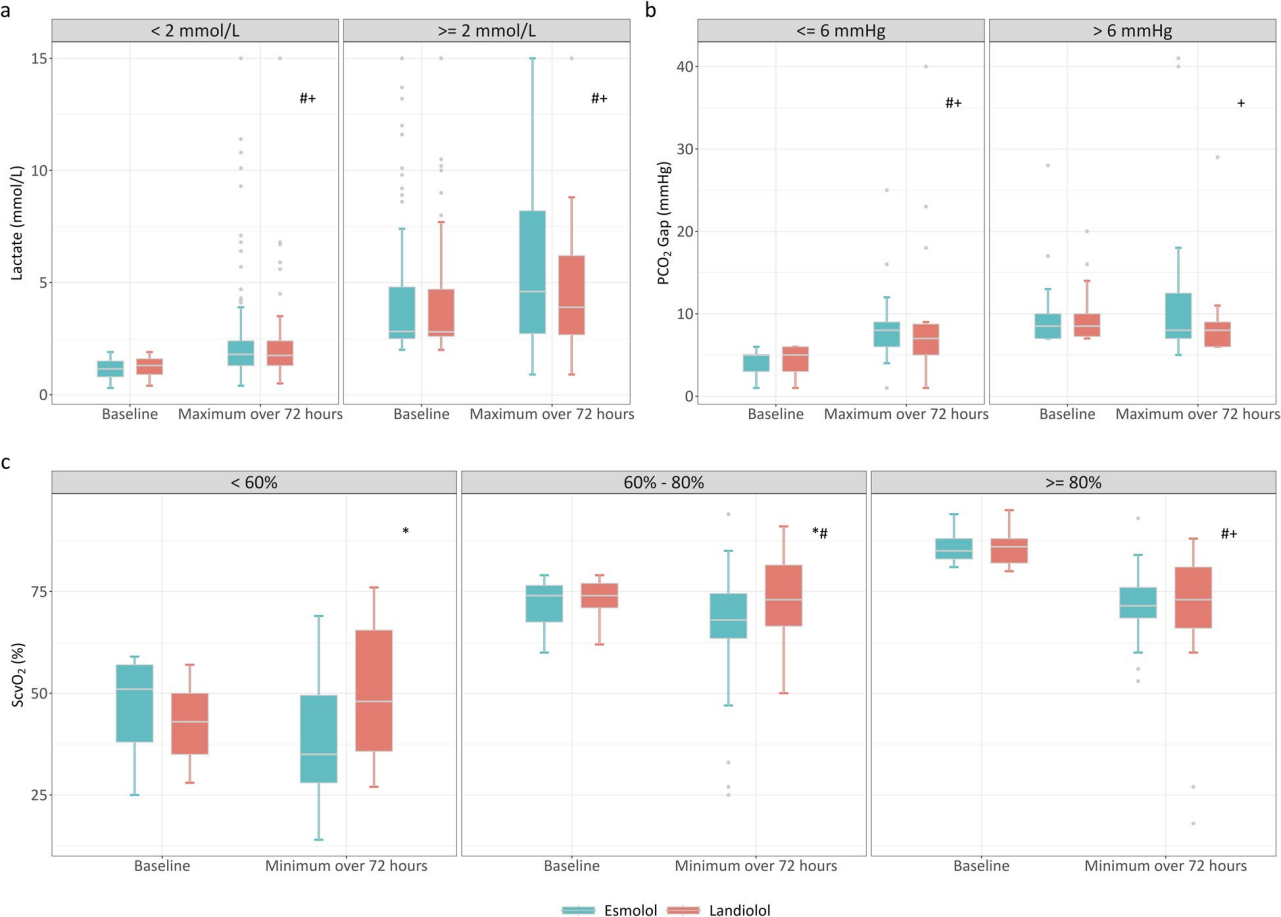
Exploratory analysis of lactate (**a**), PCO_2_ gap (**b**) and ScvO_2_ (**c**) evolution stratified by baseline levels. * indicates a significant difference between Esmolol and Landiolol group at baseline and over 72 h. # indicates a significant difference between baseline and over 72 h. in Esmolol group. + indicates a significant difference between baseline and over 72 h. in Landiolol group

Baseline and maximal lactate levels during 72 h. of β-blocker infusion were similar in the Landiolol and Esmolol groups, for baseline levels (1.7 [1.2, 2.6] mmol/L vs. 1.7 [1.0, 2.8] mmol/L, respectively, *p* = 0.416), and maximal levels (2.3 [1.5, 3.9] mmol/L vs. 2.4 [1.6, 4.5] mmol/L, respectively, *p* = 0.550) (Fig. 3a). Nevertheless, lactate levels increased significantly over 72 h. in both groups (*P* < 0.001). Notably, the magnitude of lactate increase (Δlac) was smaller in the Landiolol group compared to the Esmolol group (0.4 [−0.1, 1.3] mmol/L vs. 0.7 [0.1, 1.8] mmol/L, respectively, *p* < 0.001) (Table S4). Baseline and minimal ScvO_2_ during 72 h. were 80% (72%, 86%) and 73% (63%, 78%) in the Landiolol group, which were significantly higher than in the Esmolol group (baseline: 74% [63%, 79%], *p* < 0.001 and minimal: 67% [53%, 72%], *p* < 0.001, respectively) (Fig. 3b). There was no significant difference in the baseline PCO_2_ gap between the Landiolol and Esmolol groups (6.0 [4.0, 7.5] vs. 6.0 [4.0, 7.0] mmHg, respectively, *p* = 0.840). However, the maximal PCO_2_ gap during the first 72 h. was significantly lower in Landiolol group than in the Esmolol group (7.0 [6.0, 9.0] vs. 8.0 [6.0, 10.0] mmHg, respectively, *p* = 0.040) (Fig. 3c). Additionally, an exploratory analysis stratified by baseline levels was performed to evaluate the evolution of lactate (≥2 mmol/L, <2 mmol/L), ScvO_2_ (>80%, 60–80%, <60%), and PCO_2_ gap (>6 mmHg, ≤6 mmHg) (Fig. 4). Details are presented in Table S4 and S5.

### Outcome

ICU mortality was significantly lower in the Landiolol than in the Esmolol group. The 60-day mortality was 15% (22 out of 146) for Landiolol vs. 28% (82 out of 292) for Esmolol (*p* = 0.003). Additionally, ICULOS and HLOS were shorter in the Landiolol group compared to the Esmolol group.

### Sensitivity analysis

Of the 438 patients, 418 (95%) remained in the ICU for more than 24 h. after the initiation of β-blockers infusion, and GEE analysis of these patients showed a heart rate reduction of 5.0 (1.6, 8.4) bpm (*p* = 0.004) in the Landiolol group compared to the Esmolol group. Additionally, two different seeds were utilized, yielding heart rate differences during the 72-h. period of 4.4 (1.0, 7.8) bpm (*p* = 0.012) and 4.4 (1.0, 7.7) bpm (*p* = 0.011), respectively.

### Subgroup analysis

#### Septic shock

In the 122 patients (28%) with septic shock, there was no significant difference in the mean heart rate between the Landiolol and Esmolol groups during 72 h of β-blocker infusion (99.8 [90.1, 113.2] vs. 103.5 [90.1, 113.2] bpm, respectively, *p* = 0.257). However, at 48 h., patients who received Landiolol had a significantly lower heart rate than those who received Esmolol (92.0 [78.0, 102.0] vs. 100.5 [91.0, 113.0] bpm, respectively, *p* = 0.002) (Figure S1a).

The vasopressor stability at 24 h. was similar between the groups (67 vs. 74%, *p* = 0.411) (Table S2). During the 24-h. infusion, patients receiving Landiolol experienced a significantly larger increase in MAP compared to those receiving Esmolol (0.26 vs. −0.08 mmHg per hour, respectively, *p* = 0.022), with a comparable decrease in NE dose (10.2 × 10⁻^3^ vs. 8.6 × 10⁻^3^ μg/kg/min, respectively, *p* = 0.706) (Figure S1d, S1f). However, there were no significant differences between the two groups regarding the SAP, MAP, DAP and NE dosage at each time point (Figure S1 c–f).

The maximal lactate and PCO_2_ gap during 72 h. were similar in the Landiolol and Esmolol groups. Baseline and minimal ScvO_2_ were 79% (72%, 88%) and 71% (64%, 74%), respectively, in the Landiolol group, which was significantly higher than in Esmolol group (baseline: 74% [58%, 81%], *p* = 0.028, and minimal: 64% [47%, 70%], *p* = 0.032) (Figure S2a–c).

Among septic shock patients, ICU mortality was significantly lower in the Landiolol than in the Esmolol group. No significant difference in 60-day mortality was observed between the Landiolol and Esmolol groups (33% [14 out of 42] vs. 46% [37 out of 80], *p* = 0.169). Patients with septic shock who received Landiolol had a shorter HLOS than those who received Esmolol (21.5 [9.3, 37.8] vs. 31.0 [17.8, 54.3] days, respectively, *p* = 0.031).

**Fig. 5:**
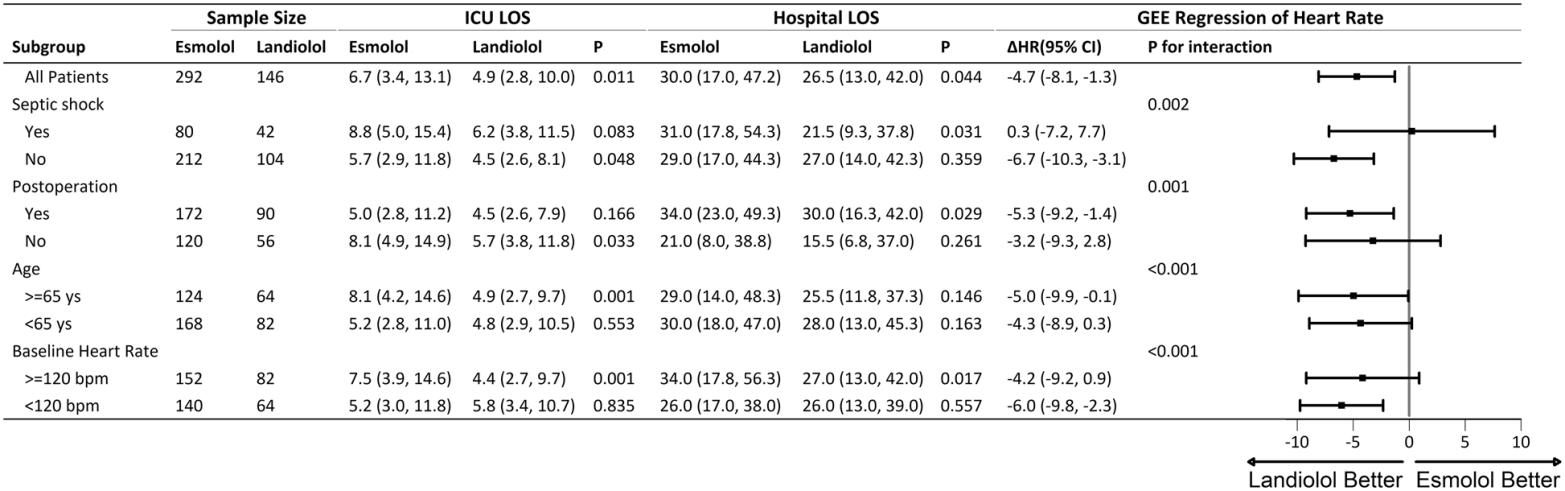
Forest plot of septic shock, post-operation, age and baseline heart rate

Subgroup analysis in non-septic shock patients, postoperative patients, elderly patients (≥65 years) and patients with a baseline heart rate <120 bpm are presented in Fig. 5. The Landiolol group exhibited a larger reduction in heart rate than the Esmolol group in these populations (Figure S3–6).

## Discussion

Our study showed that, firstly, compared to Esmolol, Landiolol decreased heart rate more efficiently in critically ill patients without increasing vasopressor requirements. Secondly, further analysis indicated that patients treated with Landiolol had significantly higher minimal ScvO_2_ levels, along with a significantly lower maximum PCO_2_ gap, suggesting that cardiac output was less affected by Landiolol than by Esmolol. Thirdly, subgroup analysis showed that in patients with septic shock, there was no significant difference in heart rate reduction between the two groups.

Several randomized control trials have assessed the effects of short-acting selective β-blockers in ICU patients [9, 10, 14, 24, 25], including those with septic shock [9]. Morelli et al. [9] conducted the first single-center study and reported that Esmolol could control the heart rate of patients with septic shock safely and effectively. However, there are certain concerns in using Esmolol in critically ill patients, especially those with cardiac dysfunction, due to its negative inotropic and blood pressure-lowering effects [4, 8]. As a ultra-short-acting β-blocker of new generation, Landiolol (its β1/β2 selectivity ratio is 255/33) was considered to have limited effects on blood pressure and cardiac function [26]. Landiolol has shown superiority over Esmolol in animal models and healthy volunteers [13, 27], but no head-to-head comparison has been conducted in ICU patients so far. Thus, there is no consensus on which β-blocker should be used due to the lack of evidence [8]. To our knowledge, the current study is the first to compare Landiolol and Esmolol regarding their therapeutic performance in critically ill patients with tachycardia.

Our results showed that Landiolol achieved superior heart rate control compared to Esmolol, without causing significant hemodynamic fluctuations during infusion. Such advantages were consistently observed in patients without septic shock, postoperative patients, elderly patients and patients with a baseline heart rate <120 bpm. However, our sub-analysis indicated that this advantage of Landiolol over Esmolol was diminished in septic shock patients. One possible explanation is that septic shock patients have less stable hemodynamic status and are more susceptible to the blood pressure-wavering effect. A recent post hoc analysis of septic shock patients with tachycardia who were treated with Esmolol suggested that those with a less vigorous arterial waveform were more likely to experience a significant reduction in cardiac output [28].

For the time being, the use of β-blockers to reduce heart rate in critically ill patients is not supported by randomized controlled trials. The recent multicenter STRESS-L study did not observe beneficial effects of Landiolol on mortality among septic shock patients [14]. Patients treated with Landiolol had higher lactate levels and NE requirements, potentially indicating a decrease in cardiac output. Similarly, the recently published Landi-SEP study also did not report improvement in clinical outcomes with Landiolol in septic shock [15]. However, these studies did not investigate the fine hemodynamic effects of Landiolol, including on ScvO_2_ [29]. This may have prevented elucidation of the mechanisms by which β-blockers exert beneficial or identification of patients in whom they may have deleterious effects [30].

Even though we did not measure cardiac output or cardiac contractility, our study provides data on the hemodynamic effects of Landiolol vs. Esmolol. Patients treated with Landiolol had significantly higher minimal ScvO_2_ levels, along with a significantly lower maximum PCO_2_ gap, compared to those treated with Esmolol. Similar results were observed in the subgroup of septic shock patients. Given the close inverse relationship between the PCO_2_ gap and cardiac output [31], it suggests that cardiac output was less affected in the Landiolol group than in the Esmolol group. In light of the significantly lower heart rate observed in the Landiolol group, this suggests that stroke volume may have been better maintained in these patients, indicating a potentially milder negative inotropic effect for Landiolol compared to Esmolol.

We acknowledge several limitations to our study. First, while the primary objective was to compare the effects of Landiolol and Esmolol on heart rate and hemodynamic variables, the study design was not powered to assess robust clinical outcomes as survival. Additionally, our cohort included patients with moderate illness severity, which may account for relatively lower mortality rate observed. Consequently, these findings should not be extrapolated to suggest cause-and-effect relationships between hemodynamic changes and clinical outcomes. Second, although PSM was employed to balance baseline characteristics, fully accounting for all potential confounders remains challenging. Selection bias is possible, given the inherent limitations of the PSM method, particularly regarding binary covariates that may not capture the full clinical spectrum. This limitation further restricts the extrapolation of hemodynamic findings to clinical outcomes. Third, our study lacked direct assessment of cardiac function, such as cardiac output or echocardiography. Nevertheless, we evaluated surrogate markers of cardiac function and tissue perfusion (ScvO_2_, PCO_2_ gap, and lactate), which may offer partial insight into cardiac performance. Fourth, the retrospective design limited our ability to reliably determine which patients experienced tachyarrhythmia specifically while receiving β-blockers. This prevented us to investigate a differential effect of β-blockers and patients with sinus tachycardia and arrhythmia. Finally, our data is limited to a single-center setting, which may restrict the generalizability of these findings.

## Conclusion

Our study indicates that, Landiolol achieves more effective heart rate control in critically ill patients with tachycardia than Esmolol, without increasing vasopressor requirements within the first 24 h. Findings from ScvO_2_ levels and PCO_2_ gap suggest that Landiolol may have a lower impact on cardiac output than Esmolol. Further studies, incorporating comprehensive hemodynamic monitoring (e.g., cardiac output, echocardiography), are warranted to clarify the clinical implications of heart rate control with β-blockers in ICU patients with tachycardia and to identify patients who may benefit from it.

## Supplementary Information

Additional file 1.

## Data Availability

The datasets used in the present study are available from the corresponding author on reasonable request.

## Abbreviations

AKI: Acute kidney injury
BPM: Beats per minute
DAP: Diastolic arterial pressure
GEE: Generalized estimating equations
HLOS: Hospital length of the stay
ICULOS: ICU length of stay
ICU: Intensive care unit
MAP: Mean arterial pressure
NE: Norepinephrine
PSM: Propensity score matching
PCO_2_ gap: Partial pressure of veno-arterial difference in carbon dioxide
PaO_2_: Partial arterial oxygen tension
FiO_2_: Fraction of inspired oxygen
SAP: Systolic arterial pressure
ScvO_2_: Central venous oxygen saturation
SOFA: Sequential Organ Failure Assessment

## References

[1] Reinelt P, Karth GD, Geppert A, Heinz G. Incidence and type of cardiac arrhythmias in critically ill patients: a single center experience in a medical-cardiological ICU. Intensive Care Med. 2001;27(9):1466–73. 11685339 10.1007/s001340101043

[2] Leibovici L, Gafter-Gvili A, Paul M, Almanasreh N, Tacconelli E, Andreassen S, Nielsen AD, Frank U, Cauda R. Relative tachycardia in patients with sepsis: an independent risk factor for mortality. QJM. 2007;100(10):629–34. 17846061 10.1093/qjmed/hcm074

[3] Shaver CM, Chen W, Janz DR, May AK, Darbar D, Bernard GR, Bastarache JA, Ware LB. Atrial fibrillation is an independent predictor of mortality in critically Ill patients. Crit Care Med. 2015;43(10):2104–11. 26154932 10.1097/CCM.0000000000001166 PMC4725582

[4] van Herpen CH, van Blokland DA, van Zanten ARH. Metabolic effects of beta-blockers in critically ill patients: a retrospective cohort study. Heart Lung. 2019;48(4):278–86. 30922521 10.1016/j.hrtlng.2019.02.004

[5] Tan S, Zhou F, Zhang Z, Wang J, Xu J, Zhuang Q, Meng Q, Xi Q, Jiang Y, Wu G. Beta-1 blocker reduces inflammation and preserves intestinal barrier function after open abdominal surgery. Surgery. 2021;169(4):885–93. 33303271 10.1016/j.surg.2020.11.004

[6] Durand M, Hagimont E, Louis H, Asfar P, Frippiat JP, Singer M, Gauchotte G, Labat C, Lacolley P, Levy B, et al. The β1-adrenergic receptor contributes to sepsis-induced immunosuppression through modulation of regulatory T-cell inhibitory function. Crit Care Med. 2022;50(9):e707–18. 35234431 10.1097/CCM.0000000000005503

[7] Bristow MR. Mechanism of action of beta-blocking agents in heart failure. Am J Cardiol. 1997;80(11a):26l–40l. 9412540 10.1016/s0002-9149(97)00846-1

[8] Guarracino F, Cortegiani A, Antonelli M, Behr A, Biancofiore G, Del Gaudio A, Forfori F, Galdieri N, Grasselli G, Paternoster G, et al. The role of beta-blocker drugs in critically ill patients: a SIAARTI expert consensus statement. J Anesth Analg Crit Care. 2023;3(1). 10.1186/s44158-023-00126-2 PMC10591347 37872608

[9] Morelli A, Ertmer C, Westphal M, Rehberg S, Kampmeier T, Ligges S, Orecchioni A, D’Egidio A, D’Ippoliti F, Raffone C, et al. Effect of heart rate control with esmolol on hemodynamic and clinical outcomes in patients with septic shock. JAMA. 2013;310(16). 10.1001/jama.2013.278477 24108526

[10] Kakihana Y, Nishida O, Taniguchi T, Okajima M, Morimatsu H, Ogura H, Yamada Y, Nagano T, Morishima E, Matsuda N. Efficacy and safety of landiolol, an ultra-short-acting β1-selective antagonist, for treatment of sepsis-related tachyarrhythmia (J-Land 3S): a multicentre, open-label, randomised controlled trial. Lancet Respir Med. 2020;8(9):863–72. 32243865 10.1016/S2213-2600(20)30037-0

[11] Iguchi S, Iwamura H, Nishizaki M, Hayashi A, Senokuchi K, Kobayashi K, Sakaki K, Hachiya K, Ichioka Y, Kawamura M. Development of a highly cardioselective ultra short-acting beta-blocker, ONO-1101. Chem Pharm Bull. 1992;40(6):1462–9. 10.1248/cpb.40.1462 1356643

[12] Ikeshita K, Nishikawa K, Toriyama S, Yamashita T, Tani Y, Yamada T, Asada A. Landiolol has a less potent negative inotropic effect than esmolol in isolated rabbit hearts. J Anesth. 2008;22(4):361–6. 19011773 10.1007/s00540-008-0640-4

[13] Krumpl G, Ulc I, Trebs M, Kadlecová P, Hodisch J. Bolus application of landiolol and esmolol: comparison of the pharmacokinetic and pharmacodynamic profiles in a healthy Caucasian group. Eur J Clin Pharmacol. 2017;73(4):417–28. 28091703 10.1007/s00228-016-2176-0

[14] Whitehouse T, Hossain A, Perkins GD, Gordon AC, Bion J, Young D, McAuley D, Singer M, Lord J, Gates S, et al. Landiolol and organ failure in patients with septic shock: the STRESS-L randomized clinical trial. JAMA. 2023;330(17):1641–52. 37877587 10.1001/jama.2023.20134 PMC10600724

[15] Rehberg S, Frank S, Cerny V, Cihlar R, Borgstedt R, Biancofiore G, Guarracino F, Schober A, Trimmel H, Pernerstorfer T, et al. Landiolol for heart rate control in patients with septic shock and persistent tachycardia. A multicenter randomized clinical trial (Landi-SEP). Intensive Care Med. 2024;50(10):1622–34. 10.1007/s00134-024-07587-1 PMC11447033 39297945

[16] von Elm E, Altman DG, Egger M, Pocock SJ, Gøtzsche PC, Vandenbroucke JP. The strengthening the reporting of observational studies in epidemiology (STROBE) statement: guidelines for reporting observational studies. Int J Surg. 2014;12(12):1495–9. 25046131 10.1016/j.ijsu.2014.07.013

[17] CSCCM, Medicine) CSoCC: Chinese guidelines for the treatment of severe sepsis/septic shock (2014). Chin J Int Med. 2015;54(6):557–81.

[18] Singer M, Deutschman CS, Seymour CW, Shankar-Hari M, Annane D, Bauer M, Bellomo R, Bernard GR, Chiche JD, Coopersmith CM, et al. The third international consensus definitions for sepsis and septic shock (sepsis-3). JAMA. 2016;315(8):801–10. 26903338 10.1001/jama.2016.0287 PMC4968574

[19] Chazot G, Bitker L, Mezidi M, Chebib N, Chabert P, Chauvelot L, Folliet L, David G, Provoost J, Yonis H, Richard JC. Prevalence and risk factors of hemodynamic instability associated with preload-dependence during continuous renal replacement therapy in a prospective observational cohort of critically ill patients. Ann Intensive Care. 2021;11(1):95. 34125314 10.1186/s13613-021-00883-9 PMC8200783

[20] Austin PC. An introduction to propensity score methods for reducing the effects of confounding in observational studies. Multivariate Behav Res. 2011;46(3):399–424. 21818162 10.1080/00273171.2011.568786 PMC3144483

[21] Kellum JA, Lameire N. Diagnosis, evaluation, and management of acute kidney injury: a KDIGO summary (Part 1). Crit Care. 2013;17(1):204. 23394211 10.1186/cc11454 PMC4057151

[22] Matthews JN, Altman DG, Campbell MJ, Royston P. Analysis of serial measurements in medical research. BMJ. 1990;300(6719):230–5. 2106931 10.1136/bmj.300.6719.230 PMC1662068

[23] Liang K-Y, Scott L. Zeger: longitudinal data analysis using generalized linear models. Biometrika. 1986;73(1):13–22.

[24] Harwood TN, Butterworth J, Prielipp RC, Royster RL, Hansen K, Plonk G, Dean R. The safety and effectiveness of esmolol in the perioperative period in patients undergoing abdominal aortic surgery. J Cardiothorac Vasc Anesth. 1999;13(5):555–61. 10527224 10.1016/s1053-0770(99)90007-1

[25] Zangrillo A, Bignami E, Noe B, Nardelli P, Licheri M, Gerli C, Crivellari M, Oriani A, Di Prima AL, Fominskiy E, et al. Esmolol in cardiac surgery: a randomized controlled trial. J Cardiothorac Vasc Anesth. 2021;35(4):1106–14. 33451954 10.1053/j.jvca.2020.12.029

[26] Domanovits H, Wolzt M, Stix G. Landiolol: pharmacology and its use for rate control in atrial fibrillation in an emergency setting. Eur Heart J Suppl. 2018;20(Suppl A):A1–3. 30188959 10.1093/eurheartj/sux037 PMC5909771

[27] Sasao J, Tarver SD, Kindscher JD, Taneyama C, Benson KT, Goto H. In rabbits, landiolol, a new ultra-short-acting beta-blocker, exerts a more potent negative chronotropic effect and less effect on blood pressure than esmolol. Can J Anaesth. 2001;48(10):985–9. 11698317 10.1007/BF03016588

[28] Morelli A, Romano SM, Sanfilippo F, Santonocito C, Frati G, Chiostri M, Agro FE, Ertmer C, Rehberg SW, Vieillard-Baron A. Systolic-dicrotic notch pressure difference can identify tachycardic patients with septic shock at risk of cardiovascular decompensation following pharmacological heart rate reduction. Br J Anaesth. 2020;125(6):1018–24. 32690246 10.1016/j.bja.2020.05.058

[29] Mantzarlis K, Vazgiourakis V, Makris D. Use of landiolol for patients with septic shock and organ failure. JAMA. 2024;331(8):705. 38411653 10.1001/jama.2023.27647

[30] Levy B, Fritz C, Piona C, Duarte K, Morelli A, Guerci P, Kimmoun A, Girerd N. Hemodynamic and anti-inflammatory effects of early esmolol use in hyperkinetic septic shock: a pilot study. Crit Care. 2021;25(1):21. 33413583 10.1186/s13054-020-03445-w PMC7791811

[31] Ltaief Z, Schneider AG, Liaudet L. Pathophysiology and clinical implications of the veno-arterial PCO(2) gap. Crit Care. 2021;25(1):318. 34461974 10.1186/s13054-021-03671-w PMC8407023

