# Supplementary material for: Comparison of the efficacy and safety of Landiolol and Esmolol in critically ill patients: a propensity score-matched study
Source: Ann Intensive Care. 2025 Jan 12;15:5. doi: 10.1186/s13613-024-01418-8 (PMC11725550; doi:10.1186/s13613-024-01418-8)
Supplement: Supplementary file 1 — Additional file 1. [file 13613_2024_1418_MOESM1_ESM.docx]

**Comparison of the efficacy and safety of landiolol and esmolol in critically ill patients: a propensity score-matched study**

Xiang Si^1,2,3#^, Hao Yuan^1,3#^, Rui Shi^1,3#^, Wenliang Song^1,3#^, Jiayan Guo^1,3^, Jinlong Jiang^1,3^, Tao Yang^1,3^, Xiaoxun Ma^1,3^, Huiming Wang^1,3^, Minying Chen^1,3^, , Jianfeng Wu^1,3*^, Xiangdong Guan^1,3*^, Xavier Monnet^2^.

1. Critical Care Medicine, The First Affiliated Hospital of Sun Yat-sen University, Guangzhou, China
2. Service de Médecine Intensive-Réanimation, Hôpital de Bicêtre, DMU CORREVE, Inserm UMR S_999, FHU SEPSIS, Groupe de Recherche Clinique CARMAS, Université Paris-Saclay, AP-HP, Le Kremlin-Bicêtre, France.
3. Guangdong Clinical Research Centre for Critical Care Medicine, 510080, Guangzhou, China

^#^XS, HY, RS and WS have contributed equally to this work.

*Correspondence:

Xiangdong Guan

Critical Care Medicine, The First Affiliated Hospital of Sun Yat-sen University, Guangzhou, China

E-mail: [guanxd@mail.sysu.edu.cn](mailto:guanxd@mail.sysu.edu.cn)

Jianfeng Wu

Critical Care Medicine, The First Affiliated Hospital of Sun Yat-sen University, Guangzhou, China

E-mail: [wujianf@mail.sysu.edu.cn](mailto:wujf@mail.sysu.edu.cn)

**Table of content**

[**Figure S1.** Trend of heart rate and hemodynamic variables during the first 72 hours among septic shock patients (Esmolol N=80, Landiolol N=42). 3](#_Toc184032932)

[**Figure S2.** Comparison of lactate levels, ScvO2, and CO2 gap at baseline and during β-blockers infusion in septic shock patients (a-c) (Esmolol N=80, Landiolol N=42) versus non-septic shock patients (d-f) (Esmolol N=212, Landiolol N=104) using raincloud plots. 4](#_Toc184032933)

[**Figure S3.** Trend of heart rate and hemodynamic variables during the first 72 hours among non-septic shock patients (Esmolol N=212, Landiolol N=104). 5](#_Toc184032934)

[**Figure S4.** Trend of heart rate during the first 72 hours among a) postoperative patients (Esmolol N=172, Landiolol N=90) and b) non-postoperative patients (Esmolol N=120, Landiolol N=56). 6](#_Toc184032935)

[**Figure S5.** Trend of heart rate during the first 72 hours among patients who were (a) older than 65 years (Esmolol N=124, Landiolol N=64) or (b) less than 65 years (Esmolol N=168, Landiolol N=82). 7](#_Toc184032936)

[**Figure S6.** Trend of heart rate during the first 72 hours among patients who had (a) baseline heart rate over 120 beats/minute (Esmolol N=152, Landiolol N=82) or (b) baseline heart rate less than 120 beats/minute (Esmolol N=140, Landiolol N=64). 8](#_Toc184032937)

[**Figure S7.** Trend of heart rate and hemodynamic variables during the first 72 hours among patients with sinus tachycardia(A, N=389) and tachyarhythmia(B, N=49). 9](#_Toc184032938)

[**Figure S8.** Trend of heart rate and hemodynamic variables during the first 72 hours among sepsis patients with sinus tachycardia(A, N=158) and tachyarhythmia(B, N=17) 10](#_Toc184032939)

[**Figure S9.** Trend of heart rate and hemodynamic variables during the first 72 hours among patients with atrial fibrillation(N=43). 11](#_Toc184032940)

[**Table S1.** Baseline characteristics before propensity score matching. 12](#_Toc184032941)

[**Table S2.** The vasopressor stability at 24th hrs after initiating β-blocker infusion 14](#_Toc184032942)

[**Table S3** The trend of heart rate, β-blocker, systolic arterial pressure, mean arterial pressure, diastolic arterial pressure, and norepinephrine dose during the first 72 hours of Esmolol and Landiolol in the whole population 15](#_Toc184032943)

[**Table S4.**  Exploratory analysis of lactate, PCO_2_ gap, and ScvO_2_ evolution stratified by baseline levels 17](#_Toc184032944)

[**Table S5**. Exploratory analysis of proportion changes in lactate, PCO_2_ gap, and ScvO_2_ stratified by different levels 18](#_Toc184032945)


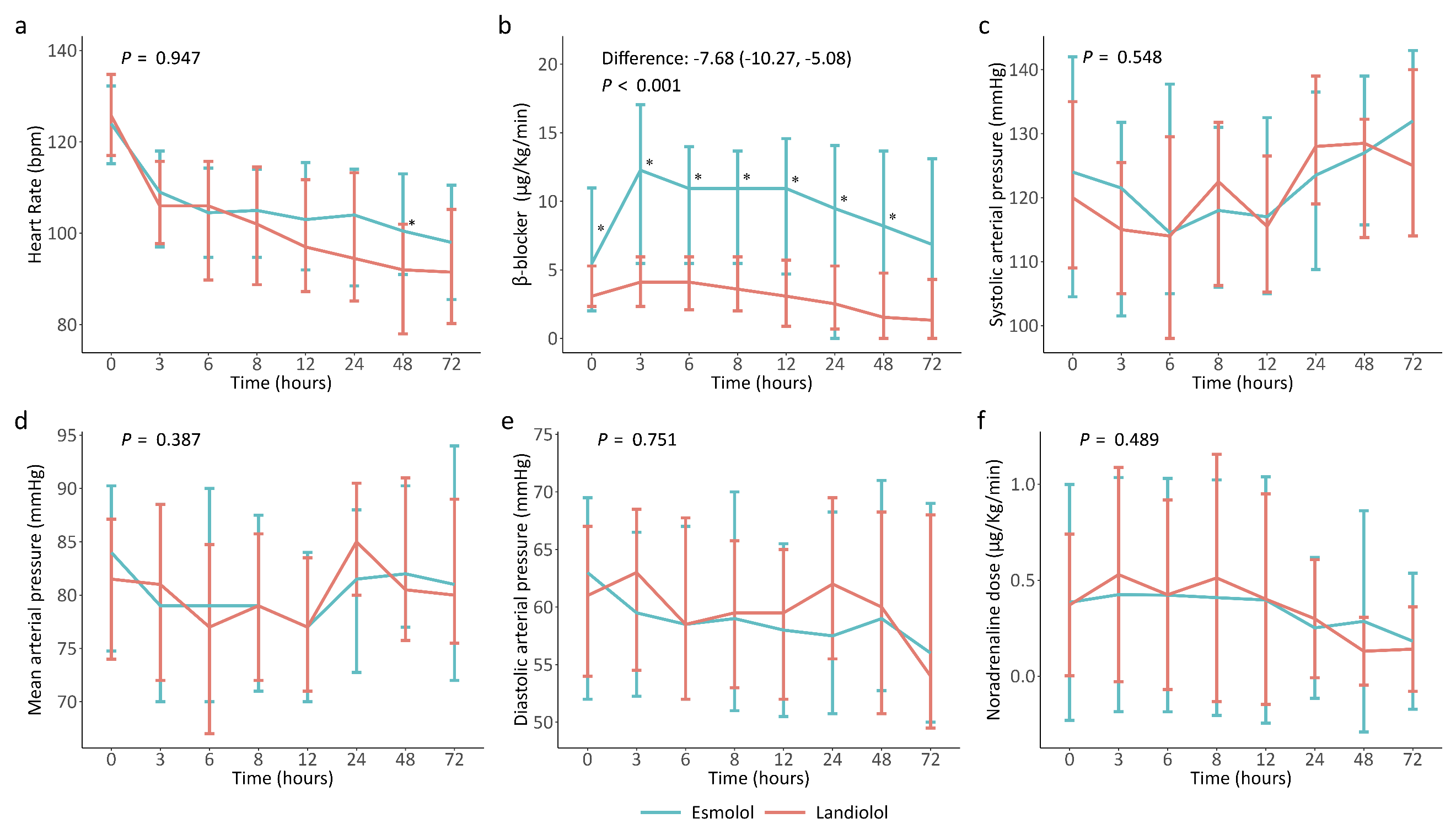


# **Figure S1.** Trend of heart rate and hemodynamic variables during the first 72 hours among septic shock patients (Esmolol N=80, Landiolol N=42).


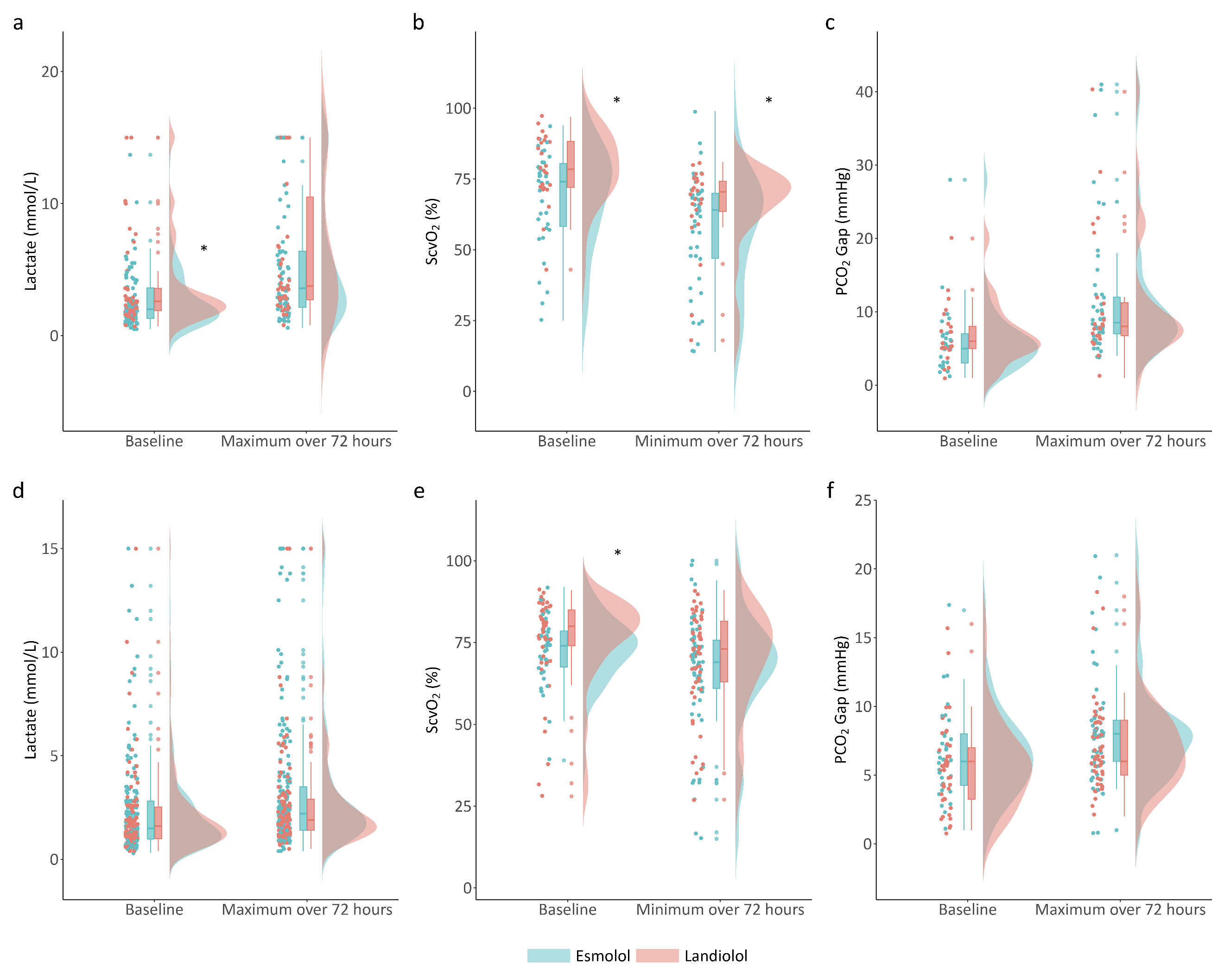


# **Figure S2.** Comparison of lactate levels, ScvO2, and CO2 gap at baseline and during β-blockers infusion in septic shock patients (a-c) (Esmolol N=80, Landiolol N=42) versus non-septic shock patients (d-f) (Esmolol N=212, Landiolol N=104) using raincloud plots.


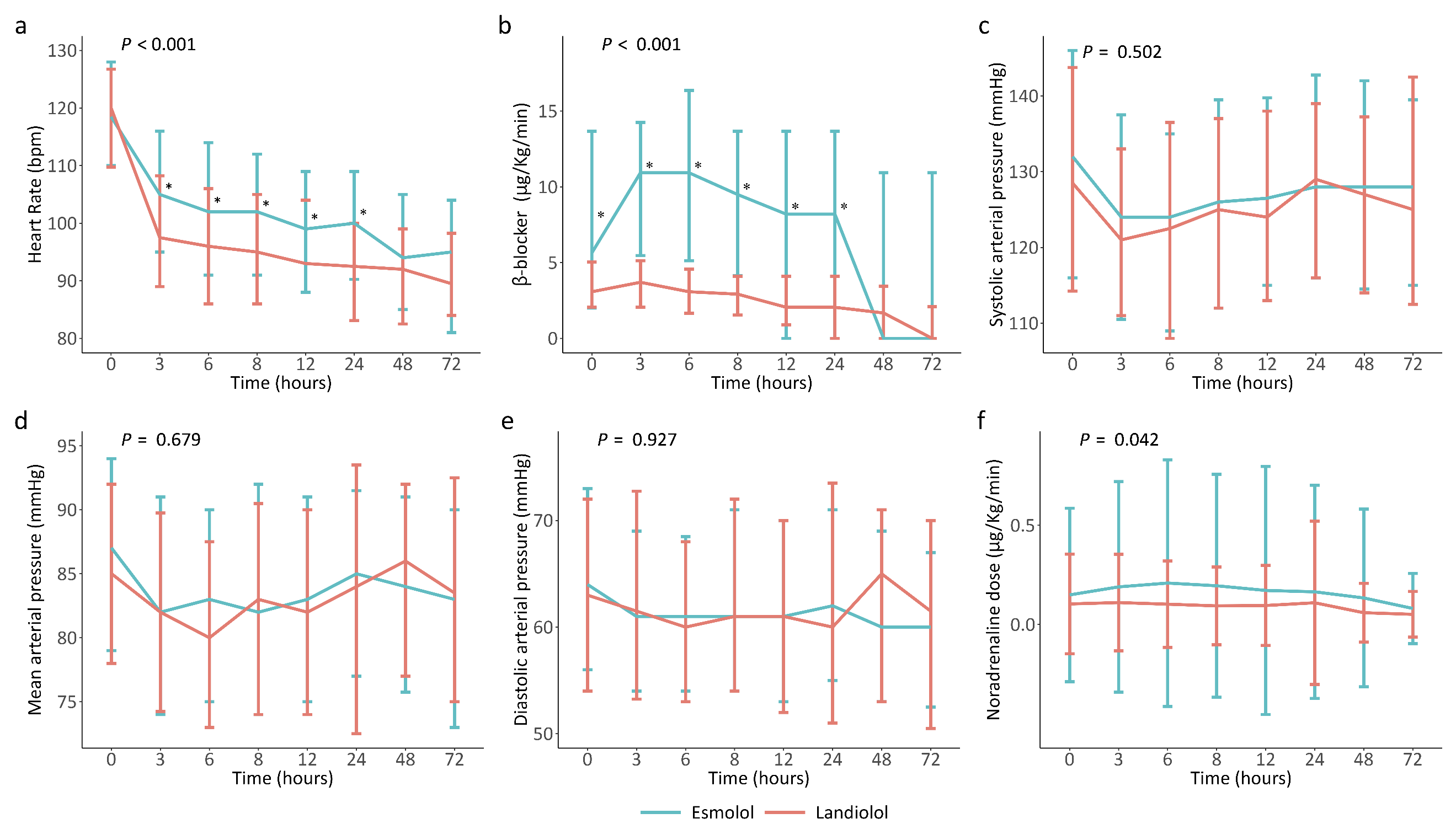


# **Figure S3.** Trend of heart rate and hemodynamic variables during the first 72 hours among non-septic shock patients (Esmolol N=212, Landiolol N=104).


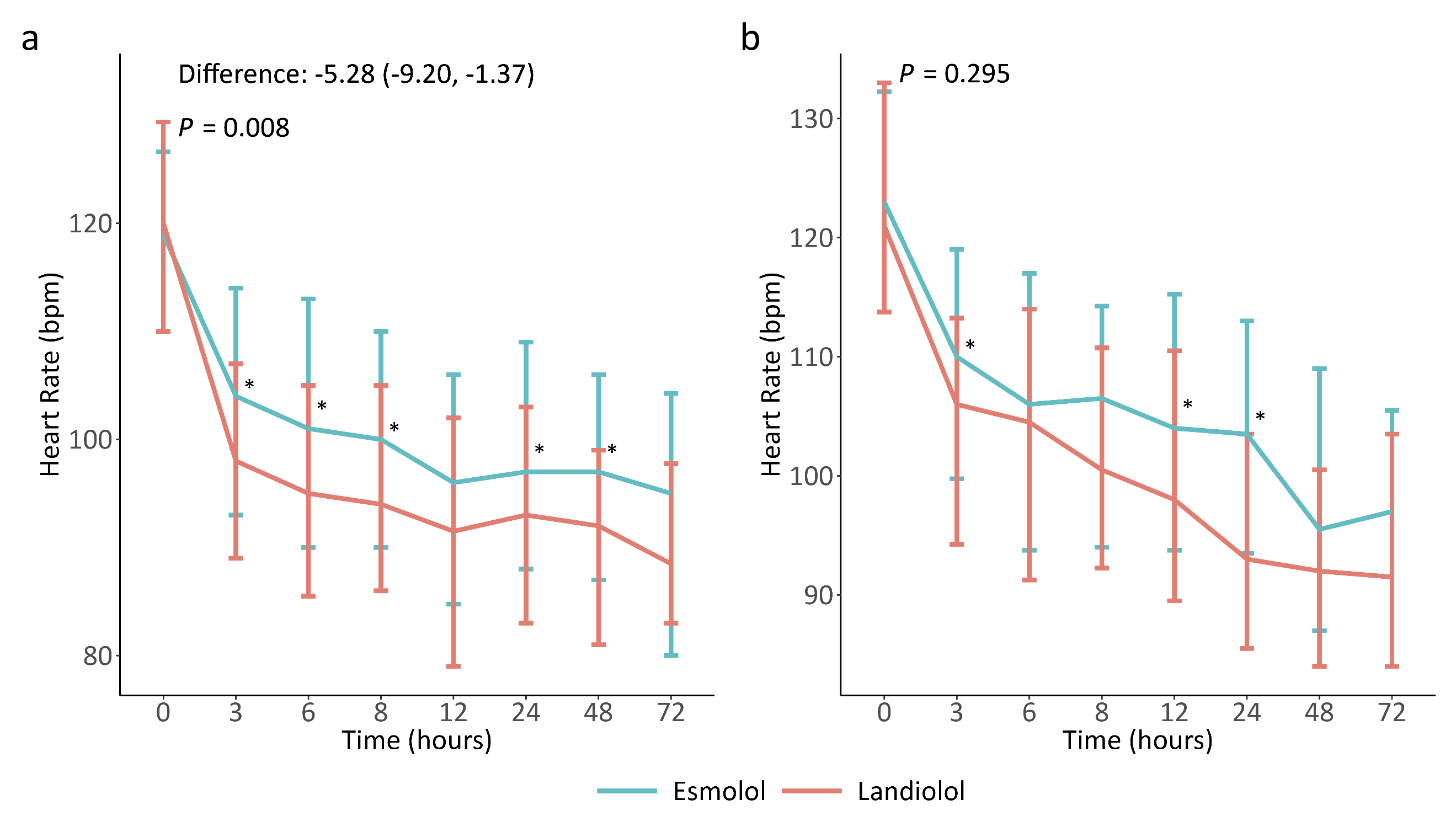


# **Figure S4.** Trend of heart rate during the first 72 hours among a) postoperative patients (Esmolol N=172, Landiolol N=90) and b) non-postoperative patients (Esmolol N=120, Landiolol N=56).


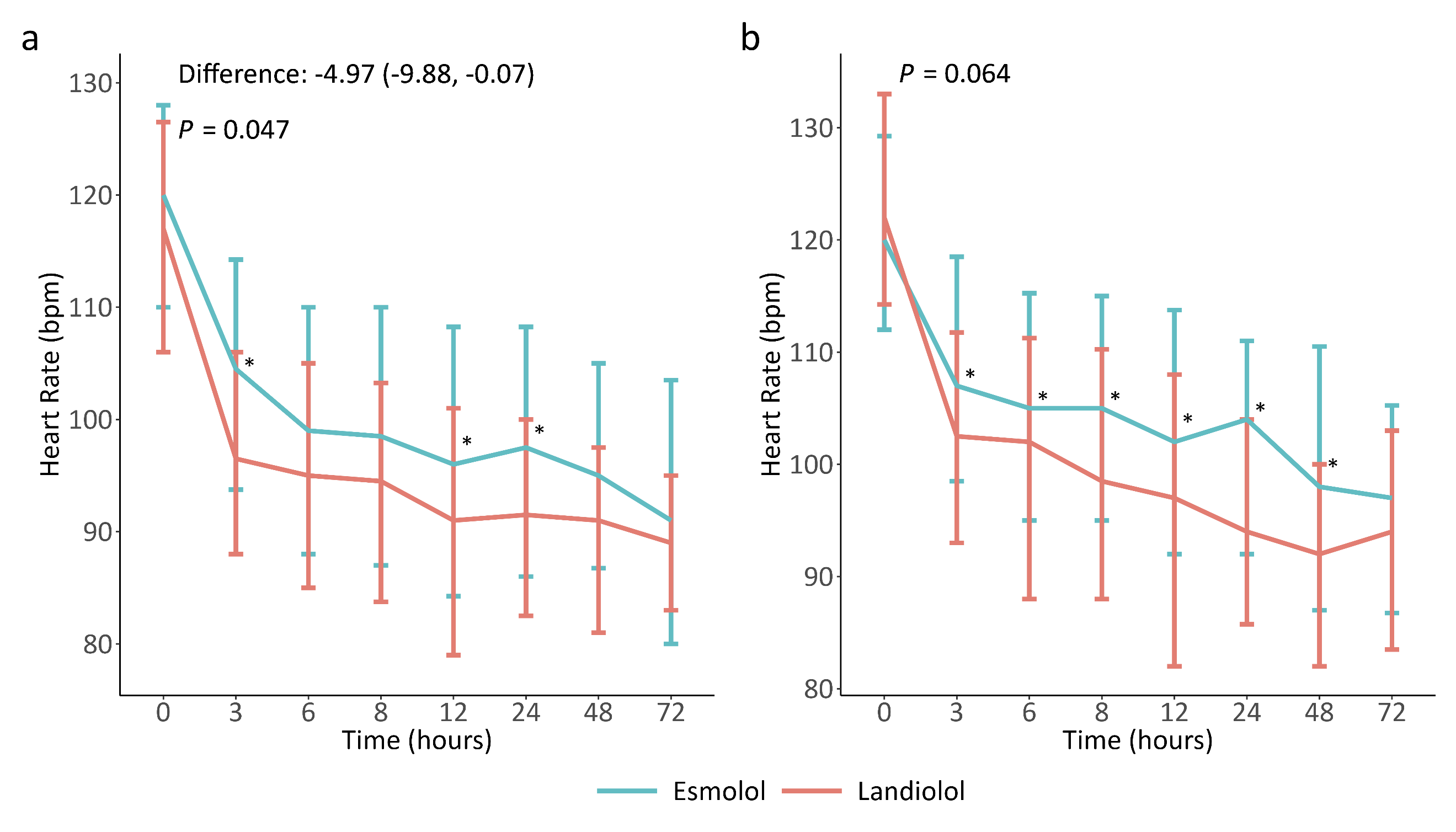


# **Figure S5.** Trend of heart rate during the first 72 hours among patients who were (a) older than 65 years (Esmolol N=124, Landiolol N=64) or (b) less than 65 years (Esmolol N=168, Landiolol N=82).


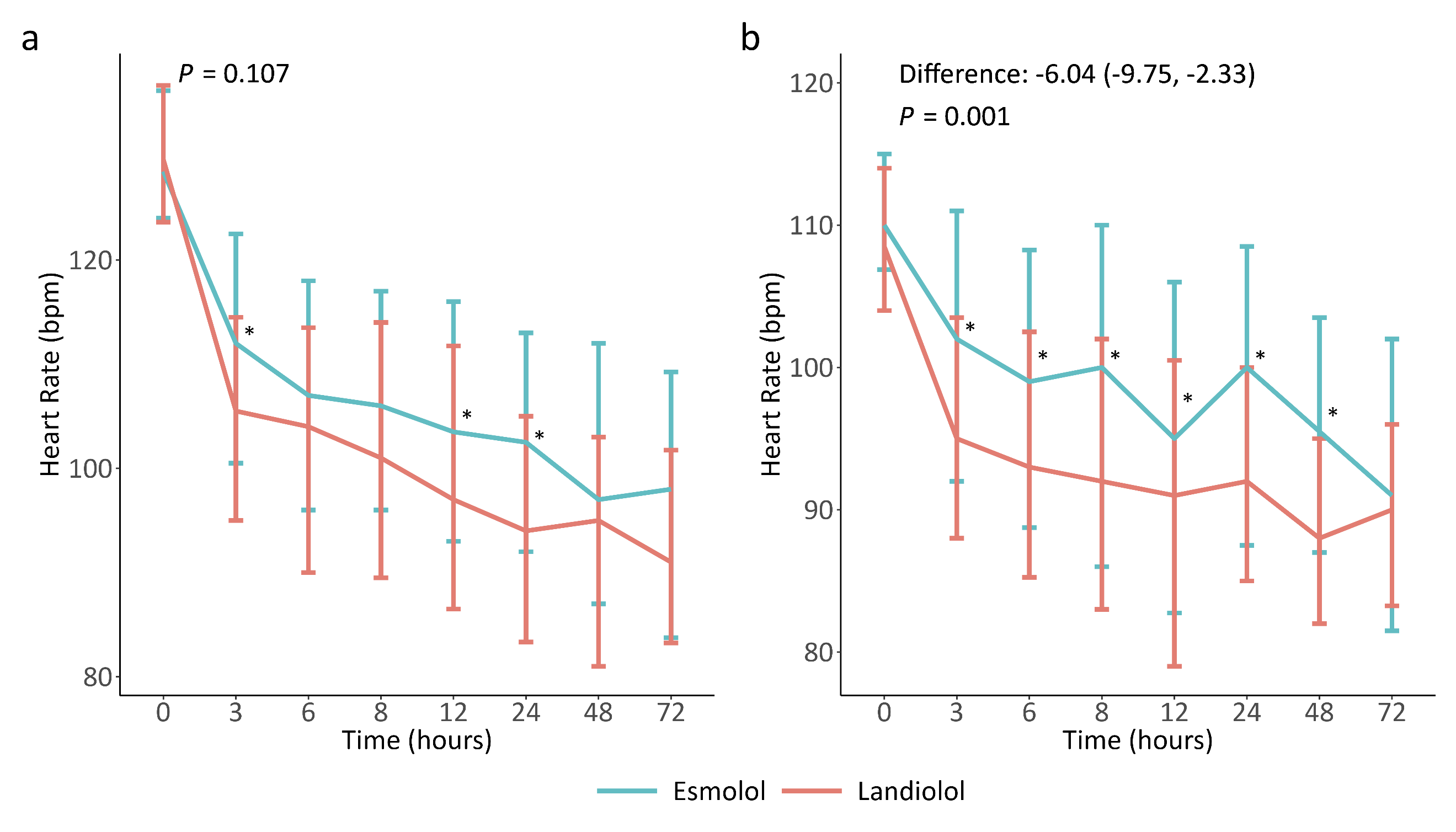


# **Figure S6.** Trend of heart rate during the first 72 hours among patients who had (a) baseline heart rate over 120 beats/minute (Esmolol N=152, Landiolol N=82) or (b) baseline heart rate less than 120 beats/minute (Esmolol N=140, Landiolol N=64).

A. Patients with sinus tachycardia (N=389)


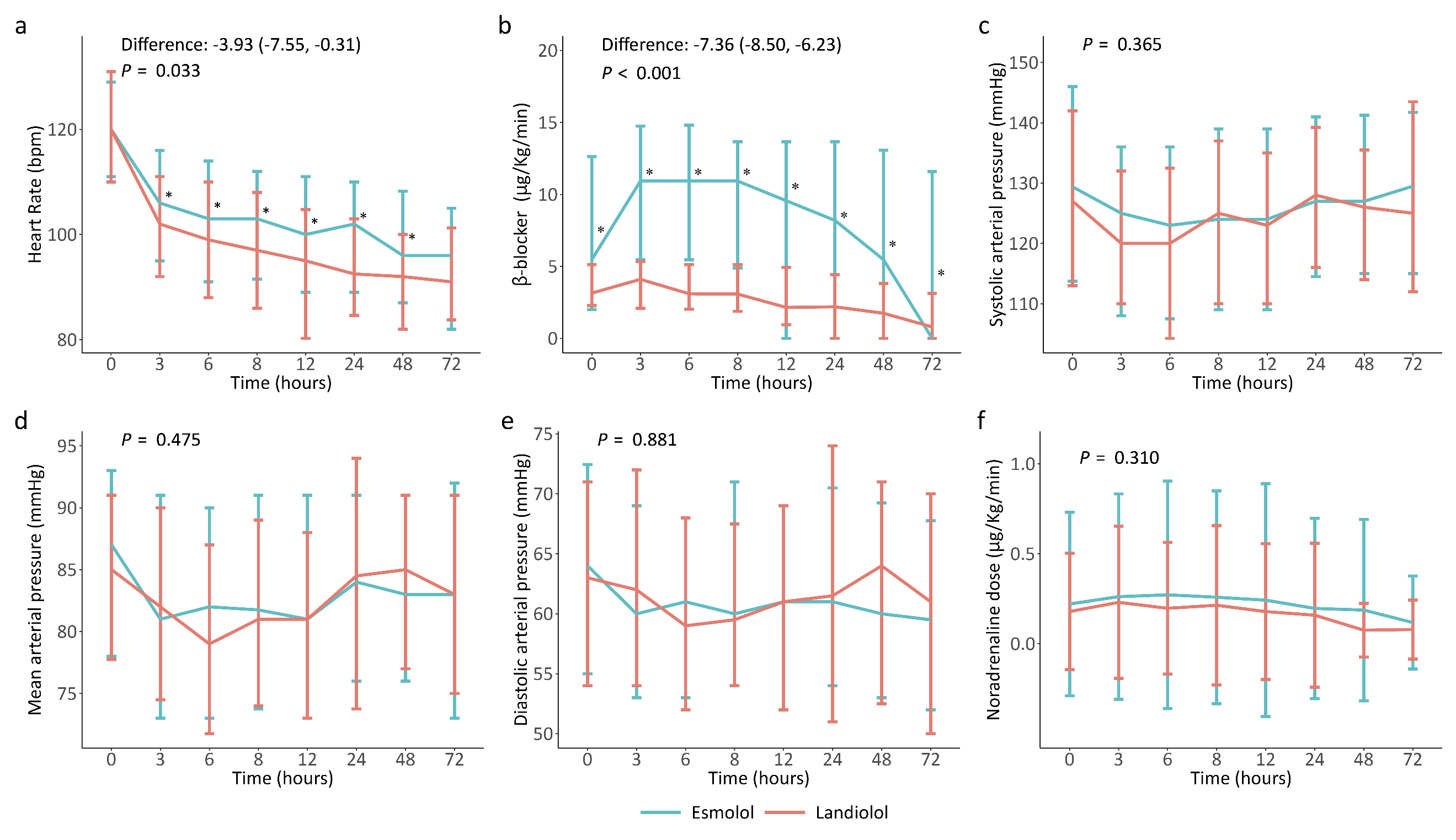


B. Patients with tachyarhythmia(N=49)


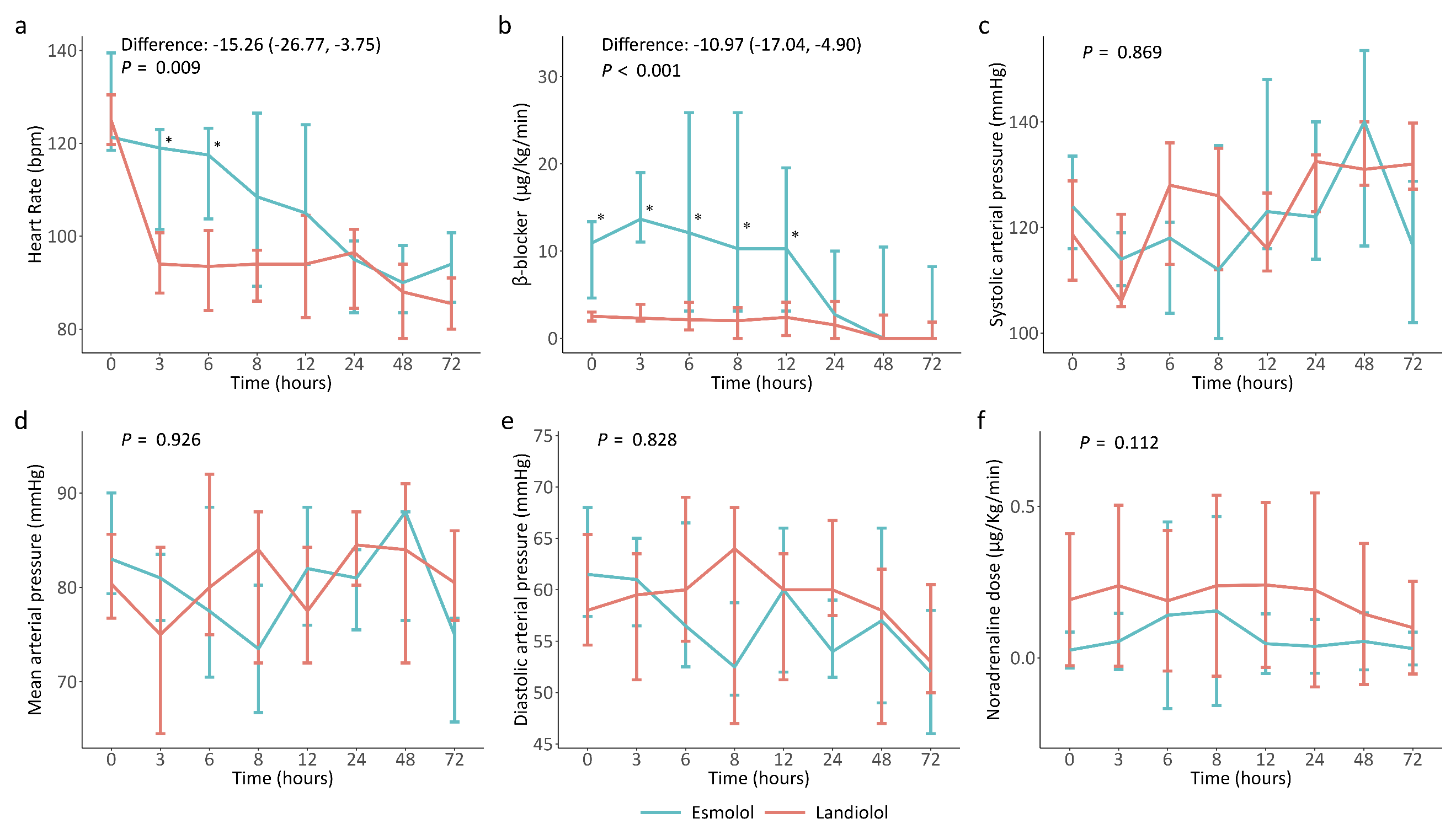


# **Figure S7.** Trend of heart rate and hemodynamic variables during the first 72 hours among patients with sinus tachycardia(A, N=389) and tachyarhythmia(B, N=49).

A. Sepsis patients with sinus tachycardia (N=158)


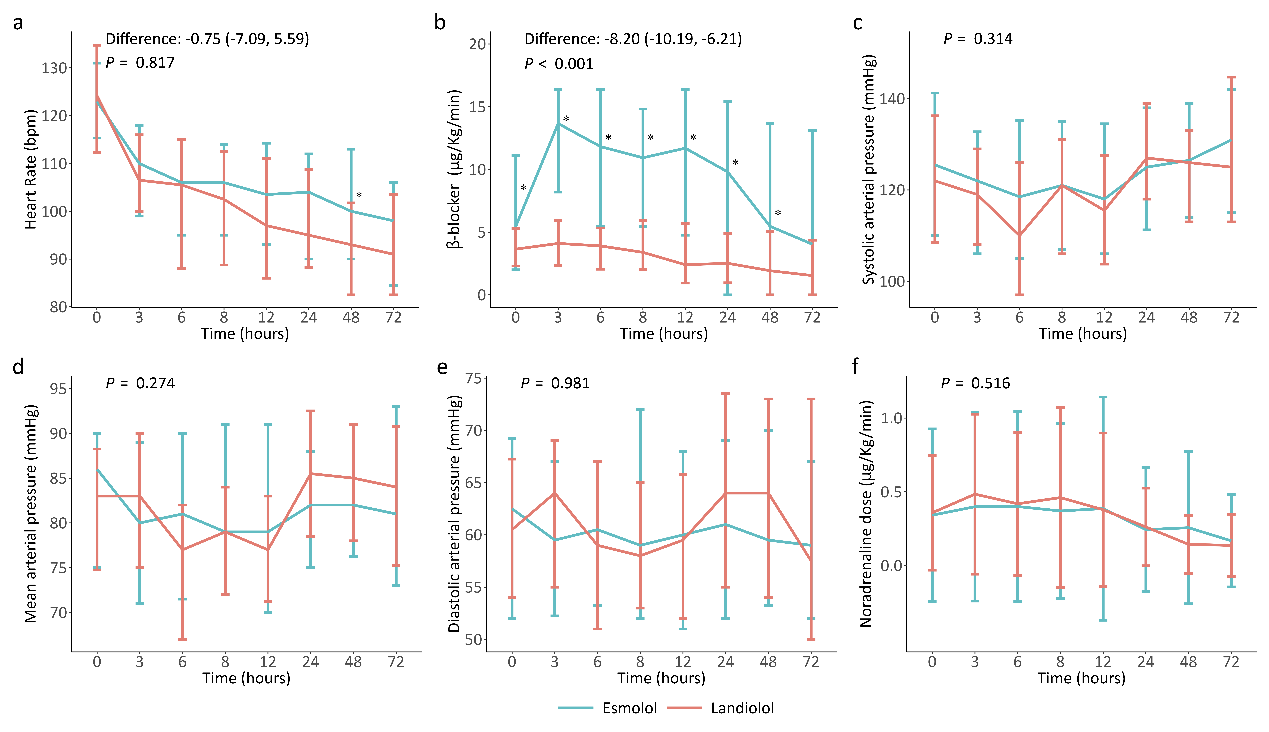


B. Sepsis patients with tachyarhythmia(N=17)


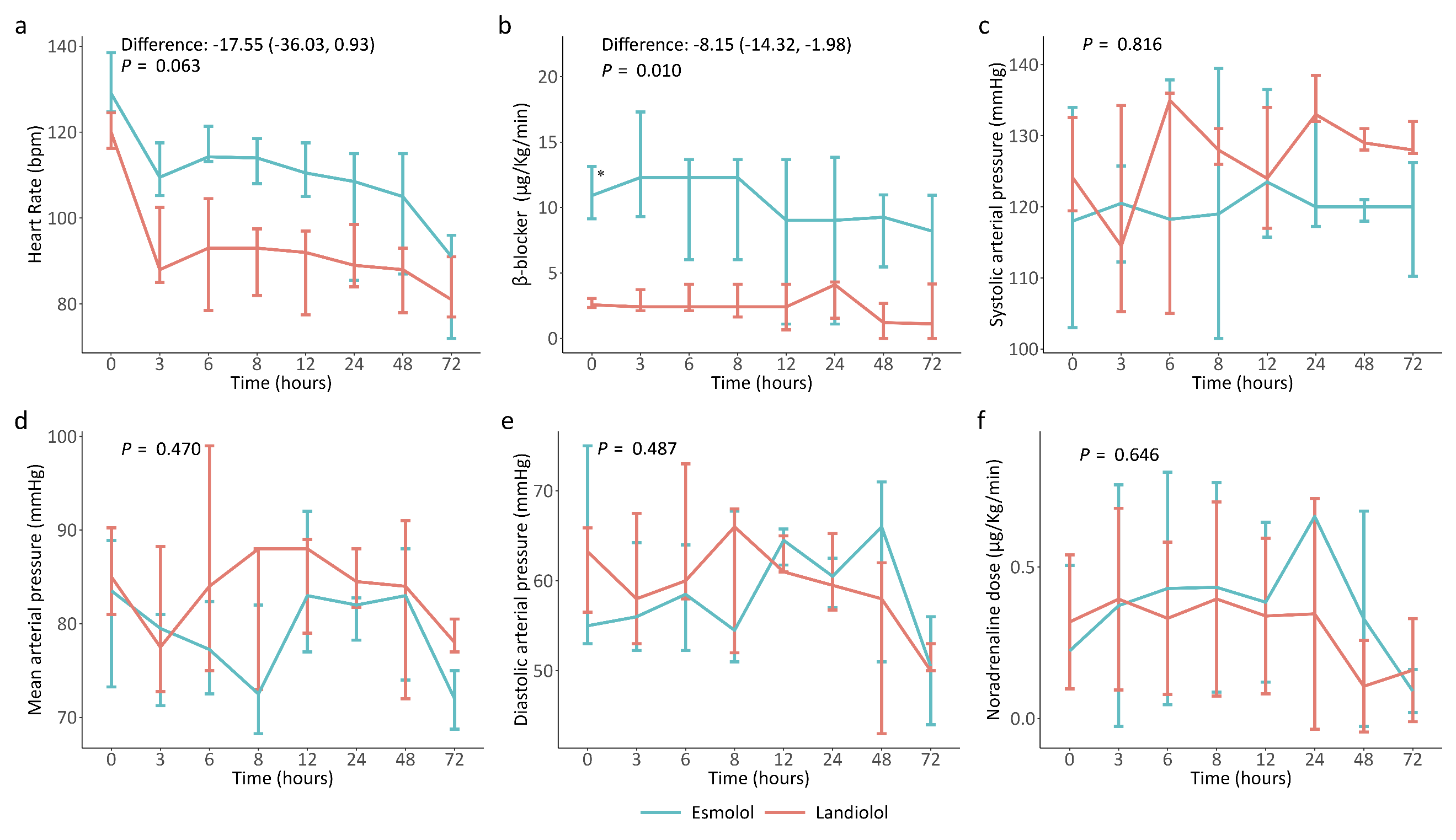


# **Figure S8.** Trend of heart rate and hemodynamic variables during the first 72 hours among sepsis patients with sinus tachycardia(A, N=158) and tachyarhythmia(B, N=17)


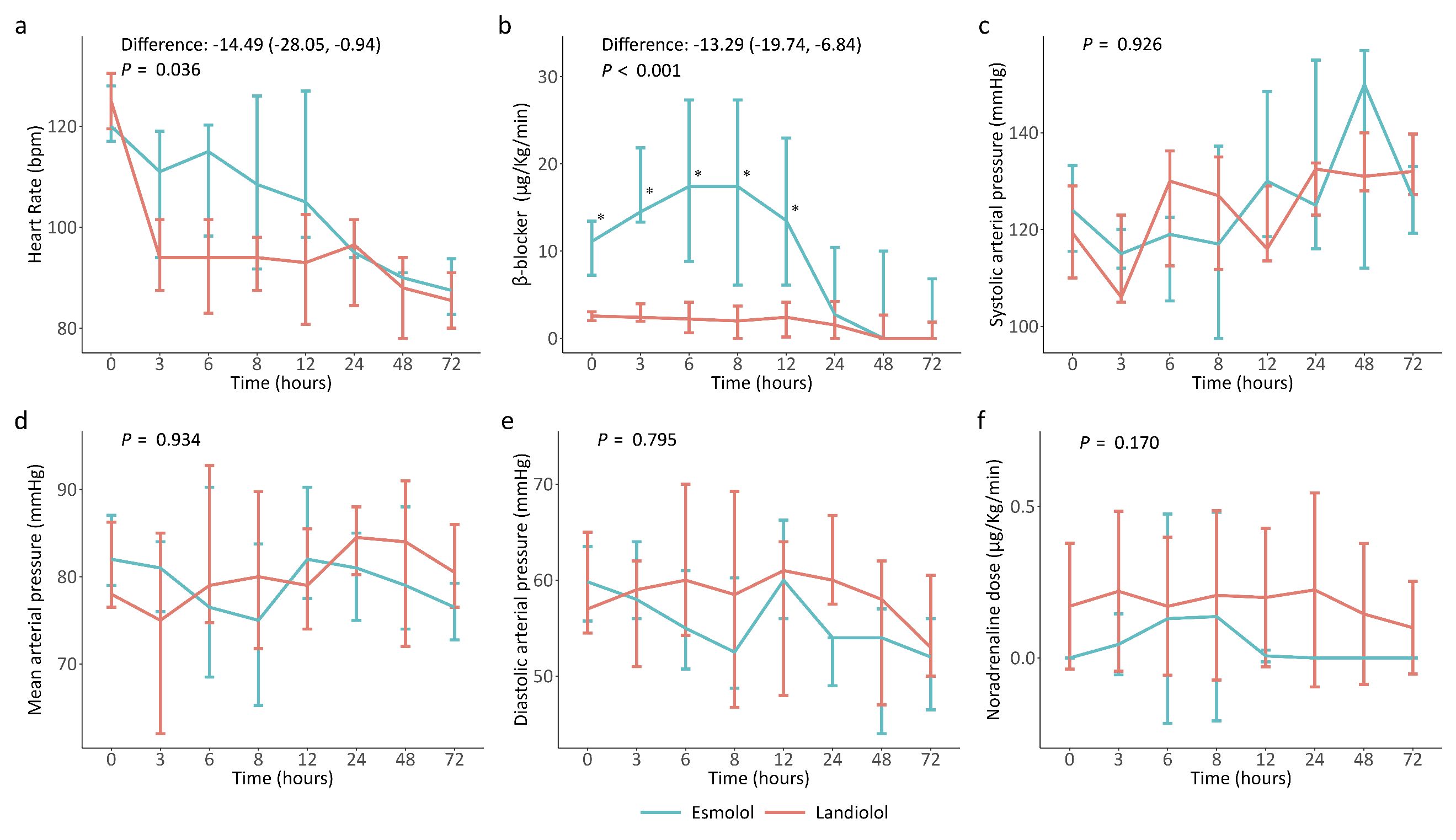


# **Figure S9.** Trend of heart rate and hemodynamic variables during the first 72 hours among patients with atrial fibrillation(N=43).

# **Table S1.** Baseline characteristics before propensity score matching.

| **Characteristic** | **Overall**, N = 2,610 | **Esmolol**, N = 2,464 | **Landiolol**, N = 146 | **SMD**^1^ | **p-value**^2^ |
| --- | --- | --- | --- | --- | --- |
| Age, median (IQR) | 57 (44.0, 67.0) | 56 (44.0, 67.0) | 61 (50.0, 71.0) | -0.33 | <0.001 |
| Female, n (%) | 864 (33.1%) | 815 (33.1%) | 49 (33.6%) | -0.01 | 0.904 |
| Primary diagnosis |  |  |  |  |  |
| Sepsis/Septic shock | 911 (34.9%) | 854 (34.7%) | 57 (39.0%) | -0.09 | 0.280 |
| Sepsis | 239 (9.2%) | 224 (9.1%) | 15 (10.3%) | -0.04 | 0.630 |
| Septic shock | 672 (25.7%) | 630 (25.6%) | 42 (28.8%) | -0.07 | 0.390 |
| Post-operative | 1,176 (45.1%) | 1,114 (45.2%) | 62 (42.5%) | 0.06 | 0.517 |
| Abdominal surgery | 427 (16.4%) | 397 (16.1%) | 30 (20.5%) |  |  |
| Trauma | 307 (11.8%) | 286 (11.6%) | 21 (14.4%) |  |  |
| Others | 442 (16.9%) | 431 (17.5%) | 11 (7.5%) |  |  |
| Medical | 523 (20.0%) | 496 (20.1%) | 27 (18.5%) | 0.04 | 0.631 |
| Acute respiratory failure | 117 (4.5%) | 103 (4.2%) | 14 (9.6%) |  |  |
| Others | 406 (15.5%) | 393 (15.9%) | 13 (8.9%) |  |  |
| Atrial fibrillation, n (%) | 302 (11.6%) | 287 (11.6%) | 15 (10.3%) | 0.04 | 0.614 |
| Hemodynamic variables |  |  |  |  |  |
| Heart rate, median (IQR) | 121.0 (113.0, 131.0) | 121.0 (113.0, 131.0) | 120.0 (110.2, 131.0) | 0.10 | 0.242 |
| MAP, median (IQR) | 87.0 (78.0, 93.0) | 87.0 (79.0, 94.0) | 85.0 (77.0, 91.0) | 0.14 | 0.021 |
| Lactate, median (IQR) | 1.9 (1.2, 2.9) | 1.9 (1.2, 3.0) | 1.8 (1.2, 2.6) | 0.08 | 0.268 |
| Norepinephrine dose, median (IQR) | 0.0 (0.0, 0.1) | 0.0 (0.0, 0.1) | 0.0 (0.0, 0.2) | -0.02 | 0.016 |
| β-blocker dose, median (IQR) | 7.1 (2.0, 13.7) | 8.2 (2.0, 13.7) | 3.1 (2.2, 5.1) | 0.86 | <0.001 |
| Time for initiating β-blocker infusion, median (IQR) | 16.8 (9.2, 30.9) | 16.7 (9.2, 30.6) | 20.8 (9.2, 35.9) | -0.19 | 0.066 |
| Subsequent oral β-blocker agent | 1,036 (39.7%) | 982 (39.9%) | 54 (37.0%) | 0.06 | 0.491 |
| Organ function |  |  |  |  |  |
| Baseline SOFA, median (IQR) | 7.7 (6.0, 9.0) | 7.7 (6.0, 9.0) | 7.7 (6.0, 9.8) | -0.01 | 0.189 |
| Mechanical ventilation, n (%) | 1,813 (69.5%) | 1,712 (69.5%) | 101 (69.2%) | 0.01 | 0.939 |
| PaO2/FiO2, median (IQR) | 305.7 (212.5, 382.5) | 305.7 (212.5, 382.6) | 297.0 (211.2, 378.1) | 0.02 | 0.656 |
| AKI stage, n (%) |  |  |  | 0.22 | 0.060 |
| 0 | 1,673 (64.1%) | 1,568 (63.6%) | 105 (71.9%) |  |  |
| 1 | 152 (5.8%) | 141 (5.7%) | 11 (7.5%) |  |  |
| 2 | 50 (1.9%) | 48 (1.9%) | 2 (1.4%) |  |  |
| 3 | 735 (28.2%) | 707 (28.7%) | 28 (19.2%) |  |  |
| Diabetes, n (%) | 460 (17.6%) | 433 (17.6%) | 27 (18.5%) | -0.02 | 0.777 |
| Hypertension, n (%) | 816 (31.3%) | 765 (31.0%) | 51 (34.9%) | -0.08 | 0.325 |
| Tumor, n (%) | 866 (33.2%) | 798 (32.4%) | 68 (46.6%) | -0.29 | <0.001 |
| ^1^Standardized Mean Difference | | | | | |
| ^2^Wilcoxon rank sum test; Pearson's Chi-squared test; Fisher's exact test  AKI, acute kidney injury; IQR, interquartile range; MAP, mean artery pressure; PaO_2_/FiO_2_, ratio of arterial oxygen tension to fraction of inspired oxygen | | | | | |

# **Table S2.** The vasopressor stability at 24th hrs after initiating β-blocker infusion

|  | **Overall**, N = 438^1^ | **Esmolol**, N = 292^1^ | **Landiolol**, N = 146^1^ | **p-value**^2^ |
| --- | --- | --- | --- | --- |
| **All Patients** | 82.2% (360/438) | 82.9% (242/292) | 80.8% (118/146) | 0.596 |
| **Septic shock** |  |  |  |  |
| Yes(n=122) | 71.3%(87/122) | 73.7%(59/80) | 66.7%(28/42) | 0.411 |
| No(n=316) | 86.4%(273/316) | 86.3%(183/212) | 86.5%(90/104) | 0.958 |
| **Postoperation** |  |  |  |  |
| Yes(n=262) | 84.4%(221/262) | 86.0%(148/172) | 81.1%(73/90) | 0.296 |
| No(n=176) | 79.0%(139/176) | 78.3%(94/120) | 80.4%(45/56) | 0.759 |
| **Age** |  |  |  |  |
| ≥65 years(n=188) | 81.4%(153/188) | 79.0%(98/124) | 85.9%(55/64) | 0.249 |
| <65 years(n=250) | 82.8%(207/250) | 85.7%(144/168) | 76.8%(63/82) | 0.081 |
| **Baseline heart rate** |  |  |  |  |
| ≥120 bmp(n=234) | 78.6%(184/234) | 80.3%(122/152) | 75.6%(62/82) | 0.407 |
| <120 bmp(n=204) | 86.3%(176/204) | 85.7%(120/140) | 87.5%(56/64) | 0.731 |
| ^1^n (%) | | | | |
| ^2^Pearson's Chi-squared test  ^2^Vasopressor stability definition: maintaining or de-escalating vasopressor doses at 24 hr compared to the treatment start | | | | |

# **Table S3** The trend of heart rate, β-blocker, systolic arterial pressure, mean arterial pressure, diastolic arterial pressure, and norepinephrine dose during the first 72 hours of Esmolol and Landiolol in the whole population

| **Characteristic** | **Overall**, N = 438 | **Esmolol**, N = 292 | **Landiolol**, N = 146 | **p-value** |
| --- | --- | --- | --- | --- |
| **Heart rate (bpm)** |  |  |  |  |
| 0 hour | 120.0 (111.0, 129.7) | 120.0 (111.0, 129.0) | 120.0 (110.3, 131.0) | 0.925 |
| 3 hour | 105.0 (94.0, 115.0) | 106.0 (95.0, 116.0) | 101.0 (90.3, 110.0) | <0.001 |
| 6 hour | 102.0 (90.0, 113.0) | 103.0 (91.0, 114.0) | 98.0 (87.0, 109.0) | 0.008 |
| 8 hour | 101.0 (89.0, 111.0) | 103.0 (91.0, 112.0) | 96.0 (86.0, 108.0) | 0.007 |
| 12 hour | 97.0 (85.0, 109.0) | 100.0 (89.0, 112.0) | 95.0 (80.0, 105.0) | 0.003 |
| 24 hour | 99.0 (87.0, 109.0) | 101.0 (89.0, 110.0) | 93.0 (84.6, 103.0) | <0.001 |
| 48 hour | 95.0 (85.0, 105.0) | 96.0 (87.0, 108.0) | 92.0 (81.8, 100.0) | 0.003 |
| 72 hour | 93.0 (83.0, 104.0) | 96.0 (82.0, 105.0) | 91.0 (83.0, 101.0) | 0.099 |
| **β-blocker (μg/Kg/min)** |  |  |  |  |
| 0 hour | 4.6 (2.0, 9.7) | 5.5 (2.0, 12.8) | 3.1 (2.2, 5.1) | <0.001 |
| 3 hour | 8.2 (3.1, 13.7) | 11.0 (5.5, 15.4) | 3.8 (2.1, 5.2) | <0.001 |
| 6 hour | 6.2 (2.2, 13.7) | 10.9 (5.5, 16.2) | 3.1 (2.0, 5.0) | <0.001 |
| 8 hour | 5.5 (2.1, 12.8) | 10.9 (4.5, 13.7) | 3.1 (1.6, 4.7) | <0.001 |
| 12 hour | 5.4 (0.6, 13.7) | 9.6 (0.0, 13.7) | 2.2 (0.9, 4.5) | <0.001 |
| 24 hour | 4.2 (0.0, 10.9) | 8.2 (0.0, 13.7) | 2.2 (0.0, 4.3) | <0.001 |
| 48 hour | 2.1 (0.0, 10.0) | 5.5 (0.0, 12.9) | 1.6 (0.0, 3.7) | 0.003 |
| 72 hour | 0.0 (0.0, 8.3) | 0.0 (0.0, 11.5) | 0.6 (0.0, 3.1) | 0.097 |
| **SAP (mmHg)** |  |  |  |  |
| 0 hour | 129.0 (113.5, 144.0) | 129.1 (114.3, 145.9) | 127.0 (110.5, 142.0) | 0.233 |
| 3 hour | 122.0 (108.0, 135.0) | 123.0 (108.0, 136.0) | 120.0 (109.0, 132.0) | 0.426 |
| 6 hour | 122.0 (106.0, 135.0) | 122.5 (107.0, 136.0) | 121.0 (105.0, 135.0) | 0.187 |
| 8 hour | 124.0 (109.0, 138.0) | 124.0 (108.0, 138.0) | 125.0 (111.0, 137.0) | 0.843 |
| 12 hour | 123.0 (110.0, 137.0) | 124.0 (110.0, 139.0) | 122.0 (110.0, 134.0) | 0.287 |
| 24 hour | 128.0 (115.0, 140.0) | 127.0 (114.3, 141.0) | 128.5 (117.0, 139.0) | 0.687 |
| 48 hour | 127.0 (114.0, 140.0) | 127.0 (115.0, 142.0) | 127.0 (114.0, 137.0) | 0.524 |
| 72 hour | 127.0 (114.0, 141.0) | 129.0 (114.8, 141.0) | 125.0 (112.0, 143.0) | 0.500 |
| **MAP (mmHg)** |  |  |  |  |
| 0 hour | 86.0 (77.2, 92.0) | 87.0 (78.0, 93.0) | 85.0 (77.0, 91.0) | 0.134 |
| 3 hour | 81.0 (73.5, 90.0) | 81.0 (73.3, 90.0) | 82.0 (74.0, 89.0) | 0.830 |
| 6 hour | 81.0 (72.0, 89.5) | 82.0 (73.0, 90.0) | 79.0 (72.0, 87.0) | 0.095 |
| 8 hour | 81.0 (73.0, 90.0) | 81.0 (73.0, 91.0) | 81.0 (74.0, 89.0) | 0.937 |
| 12 hour | 81.0 (73.0, 91.0) | 81.0 (73.5, 91.0) | 81.0 (73.0, 88.0) | 0.474 |
| 24 hour | 84.0 (76.0, 91.0) | 84.0 (76.0, 91.0) | 84.5 (75.3, 92.5) | 0.740 |
| 48 hour | 84.0 (76.8, 91.0) | 83.5 (76.0, 91.0) | 85.0 (77.0, 91.0) | 0.605 |
| 72 hour | 83.0 (73.0, 91.0) | 81.5 (73.0, 91.3) | 83.0 (75.0, 91.0) | 0.783 |
| **DAP (mmHg)** |  |  |  |  |
| 0 hour | 63.0 (54.3, 72.0) | 63.8 (55.0, 72.2) | 62.0 (54.0, 69.0) | 0.458 |
| 3 hour | 61.0 (53.0, 69.0) | 60.0 (53.0, 69.0) | 62.0 (54.0, 70.0) | 0.458 |
| 6 hour | 60.0 (52.3, 68.0) | 61.0 (53.0, 68.0) | 59.0 (52.0, 68.0) | 0.214 |
| 8 hour | 60.0 (53.0, 70.0) | 60.0 (53.0, 71.0) | 60.0 (53.0, 68.0) | 0.900 |
| 12 hour | 61.0 (52.0, 69.0) | 61.0 (52.0, 69.0) | 61.0 (52.0, 68.0) | 0.566 |
| 24 hour | 61.0 (53.8, 70.3) | 61.0 (54.0, 70.0) | 60.5 (52.0, 72.5) | 0.893 |
| 48 hour | 61.0 (53.0, 70.0) | 60.0 (53.0, 69.5) | 63.0 (52.0, 70.3) | 0.419 |
| 72 hour | 59.0 (50.0, 69.0) | 59.0 (50.8, 67.0) | 60.0 (50.0, 70.0) | 0.644 |
| **NE (μg/Kg/min)** |  |  |  |  |
| 0 hour | 0.2 (-0.2, 0.6) | 0.2 (-0.3, 0.7) | 0.2 (-0.1, 0.5) | 0.245 |
| 3 hour | 0.2 (-0.3, 0.7) | 0.3 (-0.3, 0.9) | 0.2 (-0.2, 0.6) | 0.263 |
| 6 hour | 0.2 (-0.3, 0.7) | 0.3 (-0.3, 0.9) | 0.2 (-0.2, 0.6) | 0.755 |
| 8 hour | 0.2 (-0.3, 0.7) | 0.3 (-0.3, 0.9) | 0.2 (-0.2, 0.6) | 0.693 |
| 12 hour | 0.2 (-0.4, 0.8) | 0.2 (-0.4, 0.8) | 0.2 (-0.2, 0.6) | 0.382 |
| 24 hour | 0.2 (-0.3, 0.7) | 0.2 (-0.3, 0.7) | 0.2 (-0.2, 0.6) | 0.269 |
| 48 hour | 0.2 (-0.2, 0.6) | 0.2 (-0.3, 0.7) | 0.1 (-0.1, 0.3) | 0.417 |
| 72 hour | 0.1 (-0.1, 0.3) | 0.1 (-0.2, 0.4) | 0.1 (-0.1, 0.3) | 0.462 |
|  | | | | |

# **Table S4.** Exploratory analysis of lactate, PCO_2_ gap, and ScvO_2_ evolution stratified by baseline levels

| **Characteristic** | **Overall, N = 438** | | | **Esmolol, N = 292** | | | **Landiolol, N = 146** | | |
| --- | --- | --- | --- | --- | --- | --- | --- | --- | --- |
|  | **Baseline** | **Max/Min** | **Δ** | **Baseline** | **Max/Min** | **Δ** | **Baseline** | **Max/Min** | **Δ** |
| **Lactate, Median (IQR)** | 1.7 (1.1, 2.8) | 2.3 (1.5, 4.2) | 0.6 (0.0, 1.5)^a^ | 1.7 (1.0, 2.8) | 2.4 (1.6, 4.5) | 0.7 (0.1, 1.8)^a^ | 1.7 (1.2, 2.6) | 2.3 (1.5, 3.9) | 0.4 (-0.1, 1.3)^ab^ |
| <2 | 1.2 (0.8, 1.5) | 1.8 (1.3, 2.4) | 0.6 (0.2, 1.3)^a^ | 1.2 (0.8, 1.5) | 1.8 (1.3, 2.4) | 0.7 (0.2, 1.3)^a^ | 1.3 (0.9, 1.6) | 1.8 (1.3, 2.4) | 0.4 (0.1, 1.2)^a^ |
| ≥2 | 2.8 (2.5, 4.8) | 4.2 (2.7, 7.7) | 0.6 (-0.3, 3.0)^a^ | 2.8 (2.5, 4.8) | 4.6 (2.7, 8.2) | 0.8 (-0.1, 3.6)^a^ | 2.8 (2.6, 4.7) | 3.9 (2.7, 6.2) | 0.3 (-0.7, 1.9)^a^ |
| **PcvCO_2_ Gap, Median (IQR)** | 6.0 (4.0, 7.0) | 8.0 (6.0, 9.0) | 2.0 (-1.0, 4.0)^a^ | 6.0 (4.0, 7.0) | 8.0 (6.0, 10.0) | 2.5 (-0.8, 5.0)^a^ | 6.0 (4.0, 7.5) | 7.0 (6.0, 9.0) | 1.0 (-1.0, 4.0)^a^ |
| ≤6 | 5.0 (3.0, 6.0) | 7.0 (5.0, 9.0) | 3.0 (1.0, 5.0)^a^ | 5.0 (3.0, 5.0) | 8.0 (6.0, 9.0) | 3.0 (2.0, 5.0)^a^ | 5.0 (3.0, 6.0) | 7.0 (5.0, 8.8) | 2.5 (0.0, 4.0)^a^ |
| >6 | 8.5 (7.0, 10.0) | 8.0 (6.3, 10.0) | -1.0 (-2.0, 1.0) | 8.5 (7.0, 10.0) | 8.0 (7.0, 12.5) | 0.0 (-2.0, 4.0) | 8.5 (7.3, 10.0) | 8.0 (6.0, 9.0) | -2.0 (-4.0, -1.0)^a^ |
| **ScvO_2_, Median (IQR)** | 76.0 (69.0, 83.0) | 69.0 (59.3, 76.0) | -6.0 (-13.3, 0.0)^a^ | 74.0 (63.0, 79.0) | 67.0 (53.0, 72.3) | -6.0 (-12.5, -1.0)^a^ | 79.5 (72.0, 86.0)^b^ | 72.5 (63.3, 78.0)^b^ | -7.0 (-14.8, 1.0)^a^ |
| <60% | 46.5 (37.3, 54.8) | 38.0 (32.0, 58.0) | -6.0 (-11.0, 9.0) | 51.0 (38.0, 57.0) | 35.0 (28.0, 49.5) | -7.0 (-11.0, -1.5) | 43.0 (35.0, 50.0) | 48.0 (35.8, 65.5) ^b^ | 5.5 (-0.8, 14.0) ^b^ |
| 60%-80% | 74.0 (69.0, 77.0) | 68.5 (65.0, 77.0) | -2.0 (-7.8, 1.0)^a^ | 74.0 (67.5, 76.5) | 68.0 (63.5, 74.5) | -4.0 (-8.0, 0.0)^a^ | 74.0 (71.0, 77.0) | 73.0 (66.5, 81.5) | 1.0 (-4.5, 5.5)^b^ |
| ≥80% | 86.0 (82.0, 88.0) | 73.0 (67.0, 80.0) | -13.0 (-18.0, -8.0)^a^ | 85.0 (83.0, 88.0) | 71.5 (68.5, 76.0) | -13.5 (-16.5, -9.8)^a^ | 86.0 (82.0, 88.0) | 73.0 (66.0, 81.0) | -13.0 (-18.0, -8.0)^a^ |
| Wilcoxon rank sum test was applied in Esmolol or Landiolol group  ^a^Δp<0.05; ^b^ Esmolol vs. Landiolol p<0.05 | | | | | | | | | |

# **Table S5**. Exploratory analysis of proportion changes in lactate, PCO_2_ gap, and ScvO_2_ stratified by different levels

| **Characteristic** | **Overall, N = 438** | | | **Esmolol, N = 292** | | | **Landiolol, N = 146** | | |
| --- | --- | --- | --- | --- | --- | --- | --- | --- | --- |
|  | **Baseline** | **Max/Min** | **P** | **Baseline** | **Max/Min** | **P** | **Baseline** | **Max/Min** | **P** |
| **Lactate, n (%)** | 434 | 419 | <0.001 | 289 | 275 | <0.001 | 145 | 144 | 0.003 |
| <2 | 252 (58.1) | 159 (37.9) |  | 168 (58.1) | 101 (36.7) |  | 84 (57.9) | 58 (40.3) |  |
| ≥2 | 182 (41.9) | 260 (62.1) |  | 121 (41.9) | 174 (63.3) |  | 61 (42.1) | 86 (59.7) |  |
| **PCO_2_ Gap, n (%)** | 125 | 176 | <0.001 | 62 | 101 | <0.001 | 63 | 75 | 0.013 |
| ≤6 | 79 (63.2) | 64 (36.4) |  | 38 (61.3) | 31 (30.7) |  | 41 (65.1) | 33 (44.0) |  |
| >6 | 46 (36.8) | 112 (63.6) |  | 24 (38.7) | 70 (69.3) |  | 22 (34.9) | 42 (56.0) |  |
| **ScvO_2_, n (%)** | 145 | 182 | <0.001 | 77 | 104 | 0.011 | 68 | 78 | 0.002 |
| <60% | 20 (17.8) | 46 (25.3) |  | 13 (16.9) | 32 (30.8) |  | 7 (10.3) | 14 (17.9) |  |
| 60%-80% | 74 (51.0) | 110 (60.4) |  | 47 (61.0) | 63 (60.6) |  | 27 (39.7) | 47 (60.3) |  |
| ≥80% | 51 (35.2) | 26 (14.3) |  | 17 (22.1) | 9 (8.7) |  | 34 (50.0) | 17 (21.8) |  |
| Pearson's Chi-squared test | | | | | | | | | |
